# An affinity-enhanced, broadly neutralizing heavy chain–only antibody protects against SARS-CoV-2 infection in animal models

**DOI:** 10.1126/scitranslmed.abi7826

**Published:** 2021-11-24

**Authors:** Bert Schepens, Loes van Schie, Wim Nerinckx, Kenny Roose, Wander Van Breedam, Daria Fijalkowska, Simon Devos, Wannes Weyts, Sieglinde De Cae, Sandrine Vanmarcke, Chiara Lonigro, Hannah Eeckhaut, Dries Van Herpe, Jimmy Borloo, Ana Filipa Oliveira, João Paulo Portela Catani, Sarah Creytens, Dorien De Vlieger, Gitte Michielsen, Jackeline Cecilia Zavala Marchan, George D. Moschonas, Iebe Rossey, Koen Sedeyn, Annelies Van Hecke, Xin Zhang, Lana Langendries, Sofie Jacobs, Sebastiaan ter Horst, Laura Seldeslachts, Laurens Liesenborghs, Robbert Boudewijns, Hendrik Jan Thibaut, Kai Dallmeier, Greetje Vande Velde, Birgit Weynand, Julius Beer, Daniel Schnepf, Annette Ohnemus, Isabel Remory, Caroline S. Foo, Rana Abdelnabi, Piet Maes, Suzanne J. F. Kaptein, Joana Rocha-Pereira, Dirk Jochmans, Leen Delang, Frank Peelman, Peter Staeheli, Martin Schwemmle, Nick Devoogdt, Dominique Tersago, Massimiliano Germani, James Heads, Alistair Henry, Andrew Popplewell, Mark Ellis, Kevin Brady, Alison Turner, Bruno Dombrecht, Catelijne Stortelers, Johan Neyts, Nico Callewaert, Xavier Saelens

**Affiliations:** 1 VIB-UGent Center for Medical Biotechnology, VIB, Technologiepark-Zwijnaarde 75, 9052 Ghent, Belgium.; 2 Department of Biochemistry and Microbiology, Ghent University, Technologiepark-Zwijnaarde 75, 9052 Ghent, Belgium.; 3 VIB Discovery Sciences, Technologiepark-Zwijnaarde 104B, 9052 Ghent, Belgium.; 4 KU Leuven Department of Microbiology, Immunology and Transplantation, Laboratory of Virology and Chemotherapy, Rega Institute, 3000 Leuven, Belgium.; 5 GVN, Global Virus Network, Baltimore, MD 21201, USA.; 6 KU Leuven Department of Imaging and Pathology, Biomedical MRI and MoSAIC, 3000 Leuven, Belgium.; 7 KU Leuven Department of Microbiology, Immunology and Transplantation, Rega Institute, Laboratory of Virology and Chemotherapy, Molecular Vaccinology and Vaccine Discovery Group, 3000 Leuven, Belgium.; 8 KU Leuven Department of Microbiology, Immunology and Transplantation, Translational Platform Virology and Chemotherapy (TPVC), Rega Institute, 3000 Leuven, Belgium.; 9 KU Leuven Department of Imaging and Pathology, Division of Translational Cell and Tissue Research, Translational Cell and Tissue Research, 3000 Leuven, Belgium.; 10 Institute of Virology, Medical Center University Freiburg, 79104 Freiburg, Germany.; 11 Department of Medical Imaging, In vivo Cellular and Molecular Imaging Laboratory, Vrije Universiteit Brussel, Laarbeeklaan 103, 1090 Brussels, Belgium.; 12 KU Leuven, Department of Microbiology, Immunology and Transplantation, Laboratory of Clinical and Epidemiological Virology, Rega Institute, 3000 Leuven, Belgium.; 13 Department of Biomolecular Medicine, Ghent University, 9000 Ghent, Belgium.; 14 Faculty of Medicine, University of Freiburg, 79110 Freiburg, Germany.; 15 ExeVir, Rijvisschestraat 120, 9052 Ghent, Belgium.; 16 UCB Celltech, Slough, SL1 3WE, UK.

## Abstract

Broadly neutralizing antibodies are an important treatment for individuals with coronavirus disease 2019 (COVID-19) caused by severe acute respiratory syndrome coronavirus 2 (SARS-CoV-2). Antibody-based therapeutics are also essential for pandemic preparedness against future *Sarbecovirus* outbreaks. Camelid-derived single domain antibodies (VHHs) exhibit potent antimicrobial activity and are being developed as SARS-CoV-2–neutralizing antibody-like therapeutics. Here, we identified VHHs that neutralize both SARS-CoV-1 and SARS-CoV-2, including now circulating variants. We observed that the VHHs bound to a highly conserved epitope in the receptor binding domain of the viral spike protein that is difficult to access for human antibodies. Structure-guided molecular modeling, combined with rapid yeast-based prototyping, resulted in an affinity enhanced VHH-human immunoglobulin G1 Fc fusion molecule with subnanomolar neutralizing activity. This VHH-Fc fusion protein, produced in and purified from cultured Chinese hamster ovary cells, controlled SARS-CoV-2 replication in prophylactic and therapeutic settings in mice expressing human angiotensin converting enzyme 2 and in hamsters infected with SARS-CoV-2. These data led to affinity-enhanced selection of the VHH, XVR011, a stable anti–COVID-19 biologic that is now being evaluated in the clinic.

## Camelid-derived coronavirus treatment

Strategies to treat severe acute respiratory syndrome coronavirus 2 (SARS-CoV-2) have relied upon neutralization of the virus in vivo. These strategies include treatment with antibodies, recombinant ACE2 molecules, and heavy chain–only antibodies, also known as nanobodies. In this study, Schepens *et al.* refined and improved the affinity of VHH72, a previously identified camelid-derived heavy chain–only antibody. The authors show that VHH72 neutralizes SARS-CoV-1 and SARS-CoV-2 and binds to a conserved region of the spike protein that would be difficult for larger human antibodies to access. XVR011, the clinical product that was developed through affinity enhancement of VHH72, is now being tested in the clinic.

## Teaser

A heavy chain–only antibody drug with broad SARS coronavirus neutralizing activity protects mice and hamsters.

## INTRODUCTION

Infection with severe acute respiratory syndrome coronavirus 2 (SARS-CoV-2) can lead to severe disease, characterized by dyspnea, hypoxemia, and acute respiratory distress, and can be fatal. Prophylactic vaccines for the disease caused by SARS-CoV-2, coronavirus disease 2019 (COVID-19), are gradually being rolled out in many countries and will very likely become the cornerstone of controlling the pandemic (*1*, *2*). Still, these vaccines may leave a part of the population unprotected; immunity may be short-lived and vaccine efficacy may be lower in the elderly, the age group that is most at risk of developing severe COVID-19 (*3*, *4*). Limited vaccine availability, vaccine hesitancy, and viral evolution to escape human immunity (*5*) may also compromise the impact of these vaccines. Hence, passive antibody immunotherapy with broadly neutralizing molecules to prevent or suppress viral replication in the lower airways will likely find an important place in rescuing patients with COVID-19. The early development of neutralizing antibodies by the patient correlates with avoidance of progression to severe disease (*6*), and early administration of recombinant neutralizing antibodies or high-titer convalescent plasma can avert severe disease (*7*–*9*). Regeneron’s REGN10933/REGN10987 pair and Vir’s Sotrovimab (also known as VIR-7831) recently obtained Food and Drug Administration emergency use approval for the treatment of mild to moderate COVID-19 in nonhospitalized patients who are at risk of developing severe disease (*10*, *11*). Since its first emergence in humans, however, SARS-CoV-2 variants with acquired mutations in the immunodominant receptor binding motif (RBM) have emerged. Some of these are variants of concern (VOCs) because they spread rapidly or evade host immunity. As a consequence, these VOCs display increased resistance to neutralization by convalescent plasma–derived monoclonal antibodies, including two of the three that have received emergency use approval in the United States (*5*, *12*). This reinforces the need to develop neutralizing antibodies that bind to conserved regions of the SARS-CoV-2 spike protein.

We recently reported the discovery and structural characterization of VHH72, a molecule composed of the variable domain of a heavy chain–only antibody (also known as a nanobody) with SARS-CoV-1– and SARS-CoV-2–neutralizing capacity (*13*). VHH72 binds to an epitope in the receptor binding domain (RBD) of the spike protein that is highly conserved in members of the *Sarbecovirus* subgenus of the betacoronaviruses, prevents the interaction of the SARS-CoV-1 and SARS-CoV-2 RBD with angiotensin converting enzyme 2 (ACE2), and presumably destabilizes the spike protein (*13*). We demonstrated that prophylactic administration of a prototype VHH72-human immunoglobulin G1 (IgG1) Fc domain fusion (VHH72-Fc) restricted SARS-CoV-2 replication in the lungs of experimentally infected hamsters (*14*). Although other neutralizing nanobodies against SARS-CoV-2 spike have been reported, none have reached advanced clinical development (*15*–*17*).

## RESULTS

### Protection in K18-hACE2 mice is conferred by VHH72 that binds a highly conserved region in the RBD of SARS-CoV-2

**Fig. 1.**
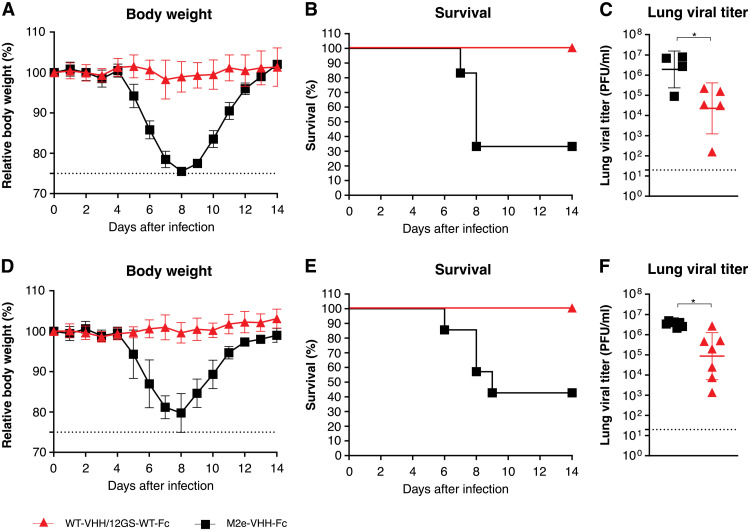
WT-VHH/12GS-WT-Fc protects K18-hACE2 transgenic mice against SARS-CoV-2 infection. (**A** to **C**) Mice received WT-VHH/12GS-WT-Fc (5 mg/kg; *n* = 17) or control M2e-VHH-Fc by intraperitoneal injection (*n* = 10). Mice were challenged 7 hours later with 8 × 10^3^ PFU of SARS-CoV-2. (A) Body weight was measured over the course of infection and plotted relative to starting body weight. Symbols represent means ± SD; *P* < 0.0001 by a two-way ANOVA. (B) Survival after challenge with SARS-CoV-2 is shown; *P* = 0.0015 by Mantel-Cox test. (C) Lung viral loads were determined on day 3 after infection from euthanized mice. *P < 0.05 by an unpaired *t* test. Data in (A) and (B) are pooled from two independent experiments. (**D** to **F**) Mice received 20 μg of either WT-VHH/12GS-WT-Fc (*n* = 15) or M2e-VHH-Fc (*n* = 14) intranasally. Mice were challenged 7 hours later with 8 × 10^3^ PFU of SARS-CoV-2, and body weight loss [(D) symbols represent means ± SD, *P* < 0.0001 by two-way ANOVA] and survival [(E) *P* = 0.0148 by Mantel-Cox test] are shown. (F) Lung viral loads were determined on day 3 after infection from euthanized mice (*n* = 7 per group). *P < 0.05 by an unpaired *t* test with Welsh correction. Symbols in (C) and (F) represent individual mice and lines indicate means ± SD. Dotted lines in (C) and (F) indicate lower limit of detection.

To obtain additional evidence of the in vivo protective potential of our broadly neutralizing VHH72-Fc prototype, we used mice expressing human ACE2 driven by the keratin 18 promotor (K18-hACE2 transgenic mice), which are susceptible to SARS-CoV-1 and SARS-CoV-2 infection (*18*, *19*). VHH72-Fc is here referred to as wild-type (WT)–VHH/12GS-WT-Fc and was produced in Chinese hamster ovary (CHO) cells (table S1). K18-hACE2 transgenic mice that were intraperitoneally injected with WT-VHH/12GS-WT-Fc (5 mg/kg) and challenged 7 hours later with a dose of 8 × 10^3^ plaque-forming units (PFU) of a clinical isolate of SARS-CoV-2 displayed no body weight loss, survived viral challenge, and had significantly reduced lung virus titers on day 3 after infection compared to control-treated animals (*P* = 0.0015 and *P* = 0,0379, respectively; Fig. 1, A to C). In addition, intranasal administration of WT-VHH/12GS-WT-Fc at a dose of 20 μg per mouse (about 1 mg/kg) protected against SARS-CoV-2–induced disease and lethality (*P* < 0.0001 and *P* = 0.0148, respectively; Fig. 1, D and E). Viral titers in the lungs were also significantly reduced in WT-VHH/12GS-WT-Fc–treated mice when compared with the controls (*P* = 0.0105; Fig. 1F). Thus, prototype molecule WT-VHH/12GS-WT-Fc protects against SARS-CoV-2 virus challenge in a prophylactic setting in both K18-hACE2 mice and hamsters (*14*).

VHH72 binds to a region in the core of the RBD that is distal from the much more variable RBM (*13*). Free energy contribution analysis by FastContact (*20*) of snapshots from Molecular dynamics simulations with the VHH72-RBD complex indicates that the epitope recognized by VHH72 has a prominent two-residue hotspot, consisting of F377 and K378, which contact VHH72 residues V100 and D100g (Kabat numbering), respectively (fig. S1A). The epitope is exposed only when the trimeric spike protein has at least one RBD in an “up” conformation (fig. S1B). In the three-RBD “down” state, the VHH72 contact region belongs to an occluded zone that makes mutual contacts with the adjacent RBDs (fig. S1C), as well as with the helix-turn-helix positioned between heptad repeat 1 and the central helix of the underlying S2 domain. These subtle inter-RBD and inter-S1/S2 contacts allow oscillation between the RBD down and the ACE2-engaging up positioning (*21*, *22*). Presumably, because of this involvement in spike conformational dynamics, the amino acid residues that contribute to the VHH72 contact region are conserved in circulating strains of SARS-CoV-2 virus (fig. S2). Moreover, deep mutational scanning analysis has shown that most mutations in the VHH72 contact region severely compromise the fold (*23*). Together, these findings support the subsequent VHH72-Fc–based drug development trajectory that resulted in the candidate anti–COVID-19 biologic XVR011 (fig. S3).

### Design and selection of a VHH72 variant with increased neutralizing activity

**Fig. 2.**
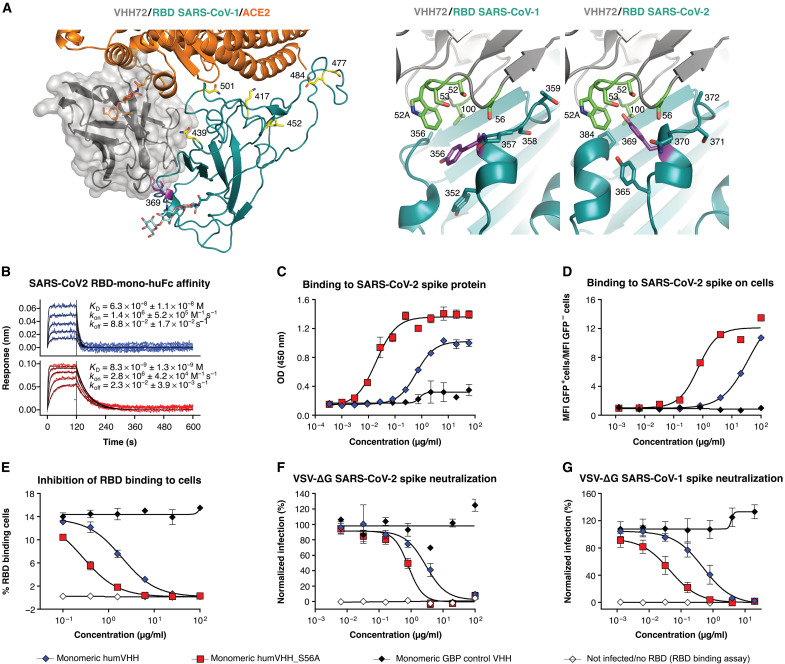
A computationally predicted VHH72 variant demonstrates enhanced neutralizing activity. (**A**) Left: Composite structure overlay of VHH72 (gray), ACE2 (orange), and SARS-CoV-2 RBD (cyan). RBD Tyr^369^, Lys^417^, Asn^439^, Leu^452^, Ser^477^, Glu^484^, and Asn^501^ are shown as sticks. ACE2 Asn^322^ and RBD Asn^343^ N-glycans are shown as orange and cyan sticks, respectively. Right: Close-up view of VHH72 bound to SARS-CoV-1 and SARS-CoV-2 RBD (cyan) based on PDB entry 6WAQ and, for SARS-CoV-2 RBD, a homology model. Residues in CDR2 and CDR3 of VHH72 and of the RBDs that are in close proximity are shown as sticks. SARS-CoV-1 RBD Tyr^356^ is oriented downward, and its counterpart Tyr^369^ in the SARS-CoV-2 RBD is oriented upward. (**B**) BLI sensorgrams of humVHH (top) and humVHH_S56A (bottom) are shown as a twofold dilution series starting at 100 nM to measure binding to immobilized SARS-CoV-2 RBD fused to a monomeric human IgG Fc. Blue and red lines represent double reference–subtracted data and the fit of the data to a 1:1 binding curve is in black. *K*_D_ is dissociation constant, *k*_on_ is on-rate, and *k*_off_ is off-rate. (**C**) humVHH and humVHH_S56A binding to SARS-CoV-2 spike as measured by an ELISA is shown. Data points indicate means ± SEM; *N* = 3. (**D**) humVHH and humVHH_S56A binding to HEK293 cell surface expressed SARS-CoV-2 spike was determined by flow cytometry. GFP fluorescence is shown normalized to the mean GFP fluorescence of noninfected and infected PBS-treated cells. GBP, GFP-binding protein (a VHH directed against GFP); MFI, mean fluorescence intensity. (**E**) Inhibition of SARS-CoV-2 RBD binding to Vero E6 cells was determined by flow cytometry (means ± SD, *N* = 3). (**F**) Neutralization of SARS-CoV-2 spike VSV pseudotypes and (**G**) SARS-CoV-1 spike VSV pseudotypes by humVHH and humVHH_S56A was measured by fluorimetry. Symbols represent means ± SD (*N* = 4).

To further enhance the potency of the prototype VHH72-Fc molecule, we applied a protein modeling–based design approach to identify a VHH72 variant with increased affinity for SARS-CoV-2 RBD through the iterative threading assembly refinement (I-TASSER) server (*24*) and Swiss-PDBViewer (*25*) on the basis of the SARS-CoV-1 RBD/VHH72 structure (Fig. 2A). Only three residues are different between SARS-CoV-2 and SARS-CoV-1 at the VHH72-RBD interface: (i) A372 (T359 in SARS-CoV-1), resulting in the loss of a glycan on N370 (N357 in SARS-CoV-1); (ii) N439 (R426 in SARS-CoV-1), resulting in the loss of an ionic interaction with VHH72 residue D61; and (iii) P384 (A371 in SARS-CoV-1). Close to P384 is Y369, for which I-TASSER predicted an upward conformation in the SARS-CoV-2 RBD-VHH72 model (Fig. 2A). The up conformation of SARS-CoV-2 Y369 sets it in a hydrophobic small cavity of VHH72, contacting complementarity-determining region 2 (CDR2) residues S52, W52a, S53, and S56 and CDR3 residue V100 (Fig. 2A). Molecular dynamics simulations with GROMACS (*26*) show that Y369 can be readily accommodated in that cavity. The later reported cryo–electron microscopy (cryo-EM) or crystal structures of SARS-CoV-2 RBD typically show Y369 in the upward conformation (*27*–*29*).

Guided by molecular modeling, we generated a set of VHH72 variants with point mutations in the residues that line the cavity that accommodates Y369 of SARS-CoV-2 RBD. These variants were rapidly prototyped in parallel as Fc fusions in the yeast *Pichia pastoris* to ensure that we would only prioritize mutations that enhanced affinity in our intended bivalent Fc fusion drug context. Introduction of S56A resulted in slower dissociation in this bivalent context and enhanced binding to SARS-CoV-2 spike expressed on the surface of 293 T cells (fig. S4). On the basis of this prototyping in *P. pastoris*, we selected VHH72 with a S56A mutation in the CDR2 for further analysis. All VHH72-Fc fusion constructs mentioned below were produced in and purified from transiently transfected CHO cells.

Next, we humanized the monovalent VHH72 and introduced the S56A mutation, and the N-terminal glutamine was replaced by a glutamic acid codon to avoid N-terminal pyroglutamate formation (fig. S5). The S56A substitution increased the affinity of the monomeric humanized VHH72 for immobilized SARS-CoV-2 RBD, by about sevenfold (Fig. 2B). Increased affinity of humVHH_S56A for SARS-CoV-2 spike was also observed in enzyme-linked immunosorbent assay (ELISA) and for SARS-CoV-2 spike expressed on the surface of mammalian cells by flow cytometry (Fig. 2, C and D). In addition, humVHH_S56A prevented the binding of SARS-CoV-2 RBD to ACE2 on the surface of Vero E6 cells seven times better than humVHH (Fig. 2E). This improved affinity correlated with significantly stronger neutralizing activity of hum_S56A (*P* = 0.0108, Fig. 2F). HumVHH_S56A also neutralized vesicular stomatitis virus (VSV) SARS-CoV-1 spike pseudotype virus with 10-fold higher potency and bound with higher affinity to SARS-CoV-1 spike and RBD than humVHH (Fig. 2G and fig. S6).

### VHH72_S56A-Fc silenced constructs with potent SARS-CoV-2 neutralizing activity

**Fig. 3.**
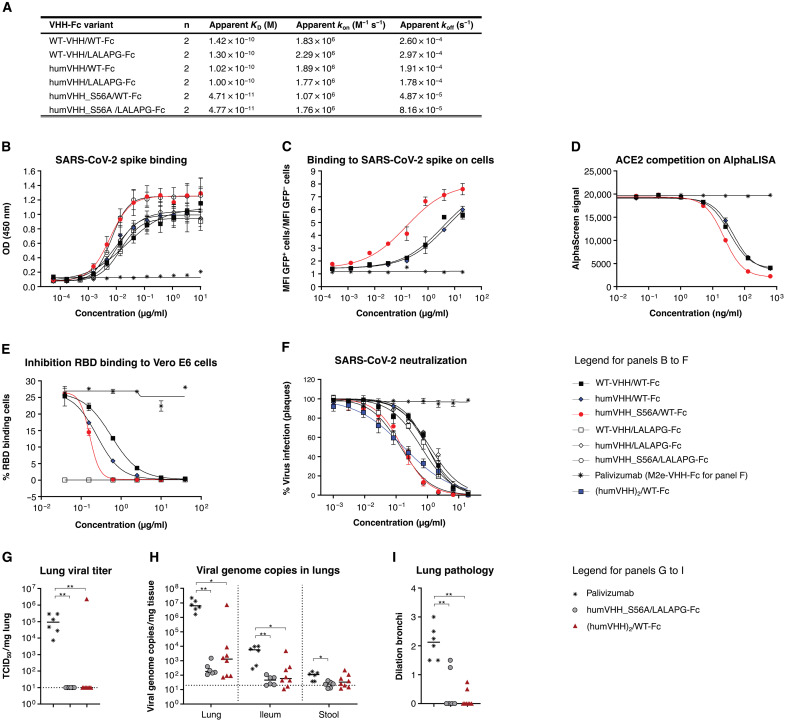
VHH72_S56A-Fc constructs have increased affinity and SARS-CoV-2 neutralizing activity. (**A**) Binding affinity of VHH72-Fc variants to immobilized SARS-CoV-2 RBD fused to mouse Fc is shown. Apparent kinetics of the 2:2 interaction were based on a global 1:1 fit of the replicate (mean, *N* = 2) data; values are the averages of replicates. (**B**) Binding of the indicated VHH72-Fc constructs to SARS-CoV-2 spike was quantified by an ELISA. Data points are means ± SD; *N* = 2. (**C**) Binding of the indicated VHH72-Fc constructs to cell surface expressed and transfected SARS-CoV-2 spike was determined by flow cytometry and shown as the ratio of the mean fluorescence intensity of the GFP-positive over GFP-negative cells. Data points are means ± SD; *N* = 2. (**D**) Inhibition of the ACE2-RBD interaction was determined by AlphaLISA. (**E**) Inhibition of SARS-CoV-2 RBD binding to Vero E6 cells by the indicated VHH72-Fc constructs was determined by flow cytometry. Data points are means ± SD; *N* = 2. (**F**) Neutralization of authentic SARS-CoV-2 by the indicated VHH72-Fc constructs. Data points in the graph represent the relative mean (± SEM, *n* = 3) number of plaques and are from one experiment that is representative of two independent replicates. (**G** to **I**) Hamsters were intraperitoneally injected with palivizumab (20 mg/kg), humVHH_S56A/LALAPG-Fc, or (humVHH)_2_/WT-Fc and challenged the next day with 2 × 10^6^ PFU of SARS-CoV-2. Infectious virus in lungs (G) and viral RNA in lungs, ileum, and stool (H) were measured on day 4 after challenge. (I) Severity score of dilated bronchi was measured on day 4 after challenge. Data were analyzed with the Kruskal-Wallis test and Dunn’s multiple comparison test (*P < 0.05 and **P < 0.01). Dotted lines in (G) and (H) indicate the lower limit of detection.

There is uncertainty about the possible contribution of IgG effector functions to disease severity in patients with COVID-19 (*30*, *31*). We opted to use the well-characterized L234A-L235A (LALA) mutations in the Fc portion of our VHH72-Fc construct, with or without the P329G mutation (L234A-L235A-P329G, LALAPG) (*32*–*34*). We validated that neither the glycine-serine linker length between the VHH and the Fc hinge (2 or 14 amino acids), nor the humanization of the VHH, nor the introduction of LALAPG mutations in the Fc affected the affinity for SARS-CoV-2 spike protein or its RBD, as determined by biolayer interferometry (BLI), ELISA, flow cytometry, or an ACE2 competition assay (Fig. 3, A to E, and fig. S7, A and B). We note that the 1:1 BLI fits of 2:2 interactions between bivalent VHH-Fcs and immobilized RBD yielded “apparent affinity constants.” Consistent with the unaltered affinity, neutralization of authentic SARS-CoV-2 virus was unaffected by these changes in the Fc region (table S1). Subsequently, we built the S56A humanized VHH variants of the WT and LALAPG-Fc fusions. We observed two- to threefold higher affinity of the VHH72-S56A-Fc constructs for immobilized bivalent SARS-CoV-2 RBD and mammalian cells expressing surface spike protein, as well as increased competition with the RBD for ACE2 binding (Fig. 3, A to E, and fig. S7). This resulted in a sevenfold enhanced potency in an authentic SARS-CoV-2 neutralization assay [for example, humVHH_S56A/LALAPG-Fc (0.13 μg/ml) compared with humVHH/LALAPG-Fc (1.22 μg/ml)] (Fig. 3F and table S1). We also increased binding valency by grafting a tandem repeat of humanized VHH72 onto human IgG1 (hIgG1) Fc, resulting in a tetravalent molecule. With this construct, we observed a greater than 100-fold higher apparent affinity for SARS-CoV-2 RBD and greater than 100-fold higher affinity binding to spike-transfected human embryonic kidney (HEK) 293S cells compared with its bivalent counterpart (fig. S7, A and C, and table S2). In line with this increased binding, the tetravalent construct displayed a 5 to 10 times more potent virus neutralizing activity compared with its bivalent counterpart (Fig. 3F; fig. S7, B and E; and table S1).

We next used the hamster SARS-CoV-2 challenge model to determine the protective potential of these potency-enhanced VHH-Fc fusion intermediate lead molecules. We assessed the protective potential of a prophylactic dose of either bivalent humVHH_S56A/LALAPG-Fc [plaque reduction neutralization test half maximum value (PRNT_50_) = 0.13 μg/ml] or tetravalent (humVHH)_2_/WT-Fc (PRNT_50_ = 0.10 μg/ml) formats (20 mg/kg), administered 1 day before challenge (Fig. 3, G to I). No infectious virus was detectable in lung homogenates from any of the VHH72-Fc–treated hamsters except for one outlier (Fig. 3G). Compared with control-treated animals, a significant reduction in viral RNA in the lungs (*P* = 0.0054 and *P* = 0.0178; >4 logs) and ileum (*P* = 0,0049 and *P* = 0.0156; 2 logs) was observed in the humVHH_S56A/LALAPG-Fc– and (humVHH72)_2_/WT-Fc–treated groups, respectively, and in stool samples for humVHH_S56A/LALAPG-Fc (*P* = 0.0273, Fig. 3H). Protection was also evident based on μ-computer tomography (μCT) imaging of the lungs on day 4 (Fig. 3I). Given the comparable protection outcome, the bivalent and tetravalent designs were both assessed further.

### Generation of a lead therapeutic

Robust expression, chemical and physical stability, and absence of atypical posttranslational modifications are important prerequisites for the “developability” of a biologic (*35*). First, to maximize the homogeneity, we truncated the upper hinge of the Fc, in common with most Fc fusions. Second, mass spectrometry analysis revealed partial C-terminal lysine removal in CHO-produced VHH72-Fc proteins, and we therefore removed this codon (fig. S8). Third, to avoid all possibility of N-terminal pyroglutamate formation and its associated charge heterogeneity, we substituted the native N-terminal glutamine residue with an aspartic acid rather than glutamic acid. Analysis of the final designs by intact (reduced) protein and peptide-level liquid chromatography–tandem mass spectrometry showed that the VHH/linker/hinge moieties were homogenous, and the Fc had CHO-typical N-glycans (fig. S9) and only well-known chemical modifications that are typical for laboratory-scale transient transfection produced antibodies: low glycation at two lysine residues (fig. S8 and table S3) and a deamidation site (fig. S10 and table S3). These chemical modifications are limited and controlled for during antibody manufacturing.

All the VHH72-Fc variants were efficiently produced in transiently transfected ExpiCHO cells with yields as high as 1.2 g/liter, after purification (table S4). The resulting humVHH_S56A/LALAPG-Fc/Gen2 construct displayed similar RBD-binding kinetics and SARS-CoV-2 neutralizing activity as humVHH_S56A/LALAPG-Fc (table S1, and fig. S7E). Combining our two potency-enhancing modalities, we also introduced the S56A mutation in each of the four VHH72 moieties in the tetravalent format in construct (humVHH_S56A)_2_/LALAPG-Fc/Gen2, which further increased the in vitro antiviral potency, reaching a PRNT_50_ value of 0.02 μg/ml (50-fold improved over the pre-lead parental construct; fig. S7E and table S1). Last, we also generated hIgG1 LALA variants of the final bivalent sequence design, with or without the S56A VHH mutation (table S1).

**Fig. 4.**
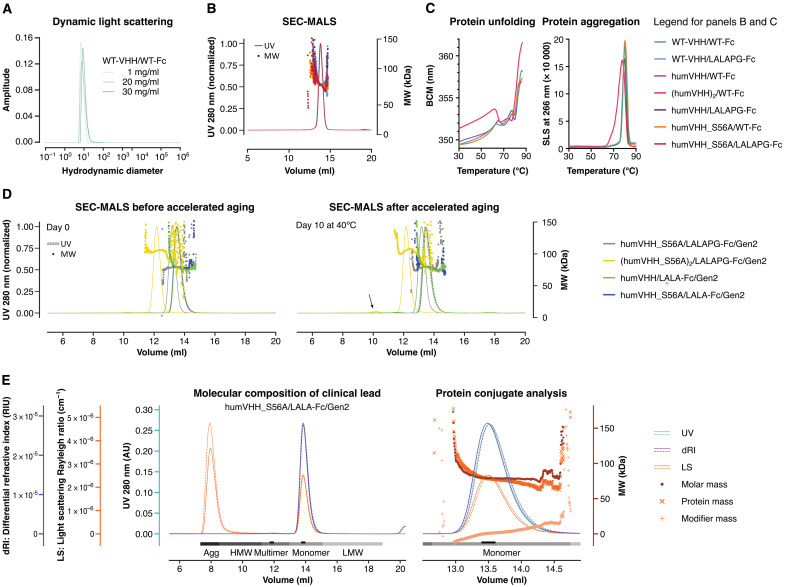
Biophysical properties of VHH72-Fc constructs. (**A**) DLS of WT-VHH/WT-Fc at 25°C is shown, at 1.0, 20, or 30 mg/ml in 25 mM histidine and 125 mM NaCl (pH 6.0). (**B**) Size exclusion chromatography–multiangle light scattering (SEC-MALS) is shown for samples (2 to 4 mg/ml) of WT-VHH/WT-Fc and variants, in which humanization, affinity-increasing (S56A), and LALAPG mutations were introduced [in 25 mM histidine and 125 mM NaCl (pH6.0)]. UV, ultraviolet 280-nm wavelength light absorption; MW, molar mass of peak fraction determined by light scattering. (**C**) Thermostability of the indicated VHH72-Fc constructs is shown. Left: Intrinsic tryptophan fluorescence as a measure of protein unfolding during a thermal ramp is expressed as barycentric mean (BCM) of the fluorescence intensity at 300 and 400 nm. Right: Protein aggregation during thermal ramp was measured by static light scattering (SLS) at 260 nm. (**D**) Ten-day storage at 40°C causes no major changes in SEC-MALS profiles of duplicate VHH72-Fc variant samples (1 mg/ml) in PBS. (**E**) SEC-MALS profiles of humVHH_S56A/LALA-Fc/Gen2 run in duplicate (solid and dashed lines) are shown. Complete peaks are indicated in grayscale for quantitative analysis; the peak apex is indicated in black for qualitative analysis. Protein conjugate analysis (calculated molar masses shown for a single replicate) was performed on the basis of the differential extinction coefficients and refractive index values of proteins versus conjugated glycan modifiers. AU, arbitrary units.

All constructs displayed good biophysical properties. Even without any formulation optimization, dynamic light scattering (DLS) analysis of WT-VHH/WT-Fc, for example, at concentrations up to 30 mg/ml in phosphate-buffered saline (PBS) revealed a homogenous sample composition, with only minor amounts of aggregation below the limit of quantification (Fig. 4A). All VHH72-Fc constructs displayed a very low tendency to multimerize or aggregate (Fig. 4B and table S5). Temperature-induced protein unfolding and aggregation was also not affected by the humanization, S56A, or LALA(PG) substitutions. Unfolding and aggregation of the tetravalent formats (humVHH)_2_/WT-Fc and (humVHH_S56A)_2_/LALAPG-Fc/Gen2, however, started at lower temperatures (Fig. 4C, fig. S11, and table S6). Even after 10 days of thermal stress at 40°C, the humanized VHH72-Fc fusions showed only a very minor tendency to aggregate (Fig. 4, D and E, and table S6). In addition, in a polyethylene glycol (PEG) precipitation assay that mimics high concentration solubility, the tetravalent molecule displayed less solubility with increasing PEG concentrations and has a longer retention time on a hydrophobic interaction column (fig. S12). The tetravalent molecule also had a more acidic isoelectric point, making it potentially less favorable for a manufacturing strategy, requiring greater optimization of ion exchange steps (table S7) (*36*).

### VHH72_S56A-Fc constructs protect hamsters against SARS-CoV-2

**Fig. 5.**
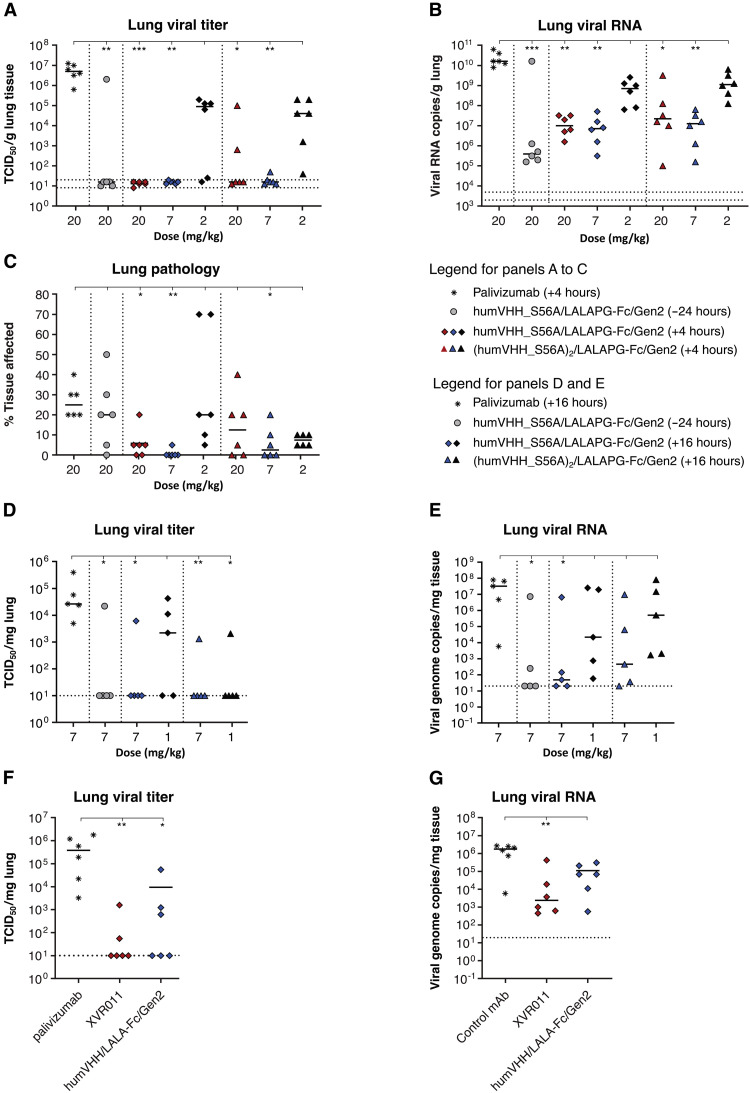
Therapeutic administration of VHH72-Fc constructs protects hamsters against SARS-CoV-2 challenge. (**A** to **C**) Hamsters were challenged with 1 × 10^4^ PFU of BetaCoV/Munich/BavPat1/2020 and 4 hours later injected intraperitoneally with humVHH_S56A/LALAPG-Fc/Gen2 or (humVHH_S56A)_2_/LALAPG-Fc/Gen2 (20, 7, or 2 mg/kg). The negative control group received palivizumab (20 mg/kg), and hamsters in a prophylactic control group received humVHH_S56A/LALAPG-Fc/Gen2 (20 mg/kg) 1 day before the challenge. Lung virus loads (A), lung viral RNA copies (B), and gross lung pathology (C) were determined on day 4 after infection. (**D** and **E**) Hamsters received an intraperitoneal injection of humVHH_S56A/LALAPG-Fc/Gen2 (7 mg/kg) 1 day before challenge or were treated by intraperitoneal injection of humVHH_S56A/LALAPG-Fc/Gen2 or (humVHH_S56A)_2_/LALAPG-Fc/Gen2 (1 or 7 mg/kg) 16 hours after infection with 2 × 10^6^ PFU of passage 6 BetaCov/Belgium/GHB-03021/2020. Palivizumab (7 mg/kg) served as a negative control. Infectious virus load (D) and viral RNA (E) were measured in the lungs on day 4 after challenge. (**F** and **G**) Hamsters were treated with palivizumab (4 mg/kg), humVHH_S56A/LALA-Fc/Gen2 or humVHH/LALA-Fc/Gen2 by intraperitoneal injection 24 hours after challenge with 2 × 10^6^ PFU of passage 6 BetaCov/Belgium/GHB-03021/2020. Infectious virus (F) and viral RNA (G) were measured in lung tissue on day 4 after infection. Data were analyzed with the Kruskal-Wallis test and Dunn’s multiple comparison test (*P < 0.05, **P < 0.01, and ***P < 0.001). Horizontal bars indicate mean. Dotted horizontal lines indicate lower limit of detection.

Next, we evaluated the in vivo efficacy of the S56A-containing fully sequence-optimized bivalent humVHH_S56A/LALAPG-Fc/Gen2 [PRNT median inhibitory concentration (IC_50_), 0.11 μg/ml] and tetravalent (humVHH_S56A)_2_/LALAPG-Fc/Gen2 (PRNT IC_50_, 0.02 μg/ml) constructs against SARS-CoV-2 challenge. HumVHH_S56A/LALAPG-Fc/Gen2 or (humVHH_S56A)_2_/LALAPG-Fc/Gen2 (doses of 20, 7, and 2 mg/kg) was administered intraperitoneally 4 hours after challenge. Control animals received palivizumab (20 mg/kg), and one group of hamsters received the bivalent construct (20 mg/kg) 1 day before challenge, as a bridge from our previous experiment (Fig. 3, G to I). In nearly all animals, virus replication in the lungs was inhibited in the prophylactic setting and in the therapeutic groups at 20 and 7 mg/kg (Fig. 5, A and B, and fig. S13A). Gross lung pathology was lowest in the animals that had been treated with the bivalent construct (7 mg/kg) (Fig. 5C). Viral titers in the nose and throat of the challenged hamsters were also significantly and dose-dependently reduced compared to the palivizumab control group [*P* = 0.001 and *P* = 0.0002 in nose and throat, respectively, for humVHH_S56A/LALAPG-Fc/Gen2 (20 mg/kg); *P* = 0.009 and 0.0075 in nose and throat, respectively, for (humVHH_S56A)_2_/LALAPG-Fc/Gen2 (20 mg/kg); *P* = 0.0454 in throat for humVHH_S56A/LALAPG-Fc/Gen2 (7 mg/kg); *P* = 0.001 and *P* = 0.0064 in nose and throat, respectively, for (humVHH_S56A)_2_/LALAPG-Fc/Gen2 (7 mg/kg)]. This indicates that parenteral, postchallenge administration of the VHH-Fc constructs restricts viral replication in the upper and lower respiratory tracts of the hamsters (fig. S13, B and C).

We independently validated this therapeutic dose finding for the bivalent and tetravalent VHH-Fc fusion lead constructs at 7 and 1 mg/kg at a different laboratory by intraperitoneal injection, 16 hours after challenge with a different SARS-CoV-2 isolate (BetaCov/Belgium/GHB-03021/2020 strain) (Fig. 5D). Infectious virus load in the lungs was significantly reduced compared to the control-treated animals for both the humVHH_S56A/LALAPG-Fc/Gen2– (*P* = 0.0153) and (humVHH_S56A)_2_/LALAPG-Fc/Gen2 (*P* = 0.009)–treated groups at 7 mg/kg (Fig. 5D). Last, we conducted a hamster challenge experiment to evaluate the in vivo impact of the S56A mutation in VHH72 in the context of humVHH_S56A/LALA-Fc/Gen2, administered at a dose of 4 mg/kg 24 hours after challenge with the BetaCov/Belgium/GHB-03021/2020 strain. Treatment with humVHH_S56A/LALA-Fc/Gen2 resulted in a stronger reduction in infectious virus and viral genomic RNA in the lungs compared to humVHH/LALA-Fc/Gen2, providing evidence that the increased in vitro affinity and antiviral potency of the S56A mutation in VHH72-Fc also resulted in enhanced protection in vivo (Fig. 5, F and G). The concentration of SARS-CoV-2 RBD VHH72-competing antibodies in hamster serum inversely correlated with the infectious virus and viral RNA loads in the lungs of the challenged hamsters (fig. S13, D and E). We prioritized the simpler bivalent design and selected humVHH_S56A/LALA-Fc/Gen2 as our clinical lead molecule, which was termed XVR011.

### XVR011 lacks off-target binding to human proteins and has reduced FcγR binding

For safe use in humans upon systemic administration, antibodies must have a low propensity for off-target binding to other human membrane/extracellular proteins. To evaluate this for XVR011, we used a human membrane protein microarray assay in which reactivity was probed against fixed HEK293 cells that each overexpress one of 5475 full-length human plasma membrane proteins and cell surface–tethered human secreted proteins and a further 371 human heterodimeric such proteins (*37*). Only four proteins were found to potentially show some binding in a high-sensitivity primary screen using XVR011 as primary antibody and an anti-hIgG1 secondary detection antibody: overexpressed proteins IgG Fc Receptor IA (FCGR1A), immunoglobulin heavy gamma-3 (IGHG3), insulin-like growth factor 1, and Calcium Homeostasis Modulator Family Member 6 (CALHM6). In a targeted confirmation experiment, we used the clinically well-validated rituximab (anti-CD20) and cells expressing its antigen as a control for potential hIgG1 Fc-mediated interactions, as well as a buffer control instead of primary antibody. Binding to the primary hits was as low or lower than that of rituximab, and a robust detection signal was only observed for fixed cells that expressed human IGHG3 chain. However, this reactivity was equally strong with the PBS and rituximab controls and was likely due to direct binding of the secondary detection antibody (fig. S14). On the basis of these results, we conclude that XVR011 is not polyreactive to human proteins, which supports its potential for safe use as a treatment for COVID-19. We also verified that the introduced LALA mutations in the context of VHH-Fc fusion construct XVR011 resulted in reduced binding to activating Fcγ receptors. Compared to rituximab, XVR011 bound to immobilized FcγRI and FcγRIIIa (V176) with a 2300- and 40-fold lower affinity, whereas binding to FcγRIIa (H167), FcγRIIa (R167), FcγRIIIa (F176), FcγRIIIb, and FcγRIIb was barely detectible and occurred with an estimated apparent equilibrium dissociation constant (*K*_D_) of more than 20 μM (fig. S15 and table S8).

### HumVHH_S56A/LALA-Fc/Gen2 binds to the RBD of a broad range of sarbecoviruses and neutralizes circulating SARS-CoV-2 variants

To test the *Sarbecovirus* binding breadth of humVHH_S56A/LALA-Fc/Gen2, we applied a flow cytometry–based yeast surface display method (*23*). HumVHH_S56A/LALA-Fc/Gen2 bound to yeast surface displayed RBD of SARS-CoV-2, GD-Pangolin, RaTG13, SARS-CoV-1, LYRa11, and WIV1 (all clade 1) with high affinity. In addition, humVHH_S56A/LALA-Fc/Gen2 could bind the RBD of Rp3 and HKU3-1 (both clade 2) and BM48-31 (clade 3), whereas CB6, which recognizes the RBM (*38*), and S309, which binds to a conserved site in the RBD core (*39*), did not bind to these RBDs (fig. S16).

**Fig. 6.**
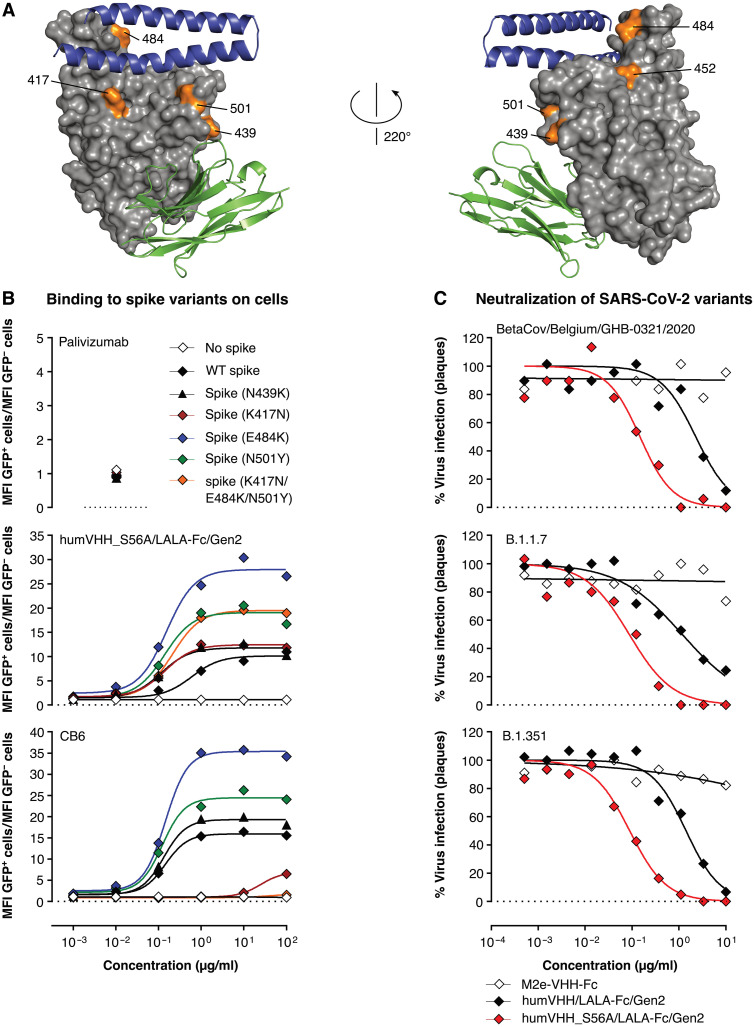
humVHH_S56A/LALA-Fc/Gen2 neutralizes SARS-CoV-2 variants of concern. (**A**) Surface view of SARS-CoV-2 RBD (gray) with VHH72 (green cartoon) and the N-terminal helixes of ACE2 (blue cartoon). The RBD-residues Lys^417^, Asn^439^, Leu^452^, Glu^484^, and Asn^501^ are indicated and marked in orange. (**B**) Binding of humVHH_S56A/LALA-Fc/Gen2, CB6, and palivizumab to SARS-CoV-1 spike was measured with the RBD replaced by WT, N439K, K417N, E484K, N501Y, or (K417N + E484K + N501Y) RBD of SARS-CoV-2 and expressed on the surface of 293 T cells. Data points represent the ratio of the MFI of transfected (GFP^+^) cells over the MFI of nontransfected (GFP^−^) cells, as determined by flow cytometry. (**C**) Neutralization of authentic SARS-CoV-2 virus determined by plaque reduction neutralization assay with threefold serial dilutions of the indicated VHH-Fc fusion constructs using BetaCov/Belgium/GHB-03021/2020, B1.1.7, or B.1.351 variant viruses.

All RBD mutations observed in the current rapidly spreading VOCs are distant from the VHH72 contact region (Fig. 6A). In a flow cytometry assay, our optimized drug lead humVHH_S56A/LALA-Fc/Gen2 showed equally strong binding to WT SARS-CoV-2 as well as to SARS-CoV-2 RBD N501Y, K417N, E484K, and K417N + E484K + N501Y SARS-CoV-2 RBD mutants expressed on the surface of mammalian cells in the context of the complete spike of SARS-CoV-1 (Fig. 6B) (*40*). N501Y is the mutation seen in both the B.1.1.7. (*41*) and B.1.351 (*42*) variants, in which the K417N and E484K are combined with it. Binding of humVHH_S56A/LALA-Fc/Gen2 to RBD variant N439K (*43*), in the periphery of the VHH72 contact region and observed 8661 times as of 2 June 2021, was also not affected (Fig. 6B). This is expected as this transition to the positively charged Lys mimics the Arg, which is present at this position in SARS-CoV-1, to which VHH72 was raised. In line with these results, humVHH_S56A/LALA-Fc/Gen2 showed equal neutralization potency against authentic SARS-CoV-2 BetaCov/Belgium/GHB-03021/2020, a B.1.1.7. variant with N501Y and a B.1.351 variant carrying K417N, E484K, and N501Y mutations (Fig. 6C).

## DISCUSSION

There remains a need for safe and effective anti–SARS-CoV-2 drugs that can prevent or treat COVID-19. Here, we report the protein engineering–based drug development of a potent cross-neutralizing, VOC-resistant anti–COVID-19 biologic. This biologic was based on a humanized, in silico affinity-enhanced VHH72 variant, which, in bivalent and tetravalent VHH-Fc format, demonstrates strong antiviral efficacy in a hamster challenge model. Compared with conventional human monoclonal antibodies, the VHH-Fc fusion construct is smaller (80 kDa versus 150 kDa) and encoded by a single gene, giving advantages with respect to dosing and manufacturability. For example, the almost two times smaller size of a VHH-Fc is a considerable advantage for subcutaneous formulations, where a very high molarity of the antibody drug needs to be provided in a small (1 to 2 ml) volume that is feasible for injection via that route. Furthermore, the single-gene–encoded nature of the VHH-Fc format, as well as its simpler homodimer assembly pathway, allows for manufacturing in alternative microbial host systems such as yeast. Such a microbial expression system comes with considerably lower cost of goods and rapid pandemic response remanufacturing opportunities.

Most of the conventional human neutralizing antibodies that are being assessed in clinical trials have been selected from convalescent repertoires. The vast majority of neutralizing antibodies in convalescent plasma are targeting the RBM in the RBD and sites on the N-terminal domain, which are hence experiencing the majority of antibody-mediated selective pressure (*44*). Expectedly, such antibodies are now affected by mutations in emerging new SARS-CoV-2 VOCs such as in strains classified in the lineages B.1.1.7, B.1.351 (*45*), and P.1 (*46*), with the latter two allowing escape from naturally acquired or spike vaccine-induced antibody neutralization. Efforts to develop a pan-*Sarbecovirus* –neutralizing antibody, such as the VHH72-based biologic described here, are therefore warranted to help protect against disease caused by the current SARS-CoV-2 pandemic virus, future SARS-CoV-2 VOCs, and potentially against future SARS-like coronavirus outbreaks.

The VHH72 discovery, determination of its complex structure bound to the SARS-CoV-1 RBD (*13*) and demonstration of its antiviral potency as Fc fusion, was first to establish that antibody binding to its non-RBM epitope region of the RBD, and, at the same time, occluding ACE2 binding to the RBM, leads to potent neutralization of sarbecoviruses such as SARS-CoV-1 and SARS-CoV-2. The functional constraints in the VHH72-binding region (inter-RBD interactions in prefusion spike, interactions with the helix-loop-helix in S2) can explain its strong conservation across the sarbecoviruses. Meanwhile, a few other monoclonal antibodies (mAbs) and VHHs, which also bind to the VHH72 core epitope (Y369, F377, and K378), have been reported (*15*, *21*, *22*, *47*–*50*), with anti–SARS-CoV-2 potency correlating with strength of competition with ACE2 binding. The much lower immunogenicity of the VHH72-binding region in humans than the epitopes on the RBM is also consistent with a recent large-scale serological survey of convalescent SARS-CoV-2 patients, which demonstrated that mAbs with epitopes strongly overlapping that of VHH72 (site II in that study) were competed against much less potently and were present in a smaller proportion of patient sera than was the case for mAbs targeting the ACE2-binding region of the RBD (*47*). These results were very similar to what was observed for S309, which is also a SARS-CoV-1/2 cross-neutralizing antibody in clinical development (*39*). Among these binding agents presently described against this “cryptic supersite,” XVR011 best combines a very strong potency both in vitro and in vivo, SARS-CoV-1 and SARS-CoV-2 cross-neutralization, very broad cross-clade *Sarbecovirus* binding, and unaltered potency against current VOCs, warranting its clinical development.

We considered it prudent in patients with progressing COVID-19 disease to mainly rely on a pure virus neutralization mechanism of action and thus to suppress Fcγ receptor binding of the antibody’s Fc domain. To silence antibody Fc-mediated effector functions, we settled on the IgG LALA-Fc mutations, which are among the best validated for this purpose (*33*). Antibodies that bind to epitopes that overlap with the epitope of VHH72 by nature already have very low Fcγ receptor activating capacity or complement-dependent cytotoxicity. Possibly, this is because this RBD region is not sterically accessible in the dominant closed spike conformation of the native spike protein, and thus, antibodies that bind to the VHH72 epitope, which requires the open spike conformation with two of the RBDs in the “up” position, may not reach a sufficiently high density to trigger Fcγ receptors or complement activation (*47*, *51*).

It was recently reported that, in a therapeutic setting, some human neutralizing antibodies require intact Fc effector functions to control SARS-CoV-2 replication in the K18-hACE2 transgenic mouse and hamster challenge models (*52*). In contrast to that report, we found that Fc effector silent humVHH_S56A/LALA(PG)-Fc/Gen2 administered 4, 16, or 24 hours after viral challenge resulted in a very strong reduction of lung viral RNA and infectious virus. This suggests that the non-RBM binding mode of neutralization, likely including spike destabilization, perhaps combined with a faster biodistribution compared with a conventional antibody, allows for efficacious viral control (*53*). The requirement of effector functionality for therapeutic efficacy appears to be very antibody dependent even with human antibodies, as a recent study also demonstrated therapeutic efficacy in the same hamster model of other RBM-binding LALAPG-modified antibodies (*54*).

Our study has several limitations. First, although the binding region of VHH72 in SARS-CoV-2 RBD is highly conserved, suggesting a functional constraint for this part of the RBD, in vitro escape selection experiments are needed to estimate the likelihood of the emergence and fitness of mutant viruses with reduced susceptibility to XVR011. Second, although we demonstrated in vivo protection against challenge with three different SARS-CoV-2 virus strains, these were all derived from clinical isolates that circulated early in the pandemic. Last, the safety and effectiveness of XVR011 in patients with COVID-19 has not yet been demonstrated. Healthy volunteer phase 1a trial and a phase 1b/2 trial in patients with moderate disease severity are presently ongoing to address this question.

The pharmaceutically fully developed VHH72-based biologic named XVR011, which combines potent neutralizing activity with high stability, broad coverage, and silenced Fc effector functionality for enhanced safety, has now completed Current Good Manufacturing Practice manufacturing and formal preclinical development. Clinical studies are now being started to evaluate safety and efficacy of rapid administration upon hospitalization of patients within the first week of COVID-19 symptoms (NCT04884295). In conclusion, XVR011 represents a promising antibody-based countermeasure against disease caused by SARS-CoV-2 and potential future zoonotic outbreaks with related sarbecoviruses.

## MATERIALS AND METHODS

### Study design

This study was designed to generate a candidate VHH72-Fc–based biologic, which is ready for clinical development. We used structure-guided molecular modeling combined with a rapid yeast-based screening approach to identify VHH72-Fc derivatives with enhanced affinity for SARS-CoV-2 RBD. Tandem repeats of VHH72 fused to hIgG1 Fc were generated as an alternative strategy to increase the affinity for SARS-CoV-2 RBD. VHH72 framework regions were humanized, linkers between the VHH and hIgG1 Fc were optimized, and we opted for an Fc domain with mutations that reduce Fc effector functions and CHO cells for recombinant protein expression. Mass spectrometry, DLS, a PEG protein solubility assay and hydrophobic interaction chromatography analysis were performed to select the VHH-Fc candidates with optimal physico-chemical properties. Lack of nonspecific antigen binding was demonstrated using a human membrane protein microarray assay, and surface plasmon resonance was used to show reduced FcγR binding of the mutated Fc. Candidate VHH72-Fc derivatives for further development were also compared in in vitro virus neutralization assays using VSV pseudotyped with SARS-CoV-1 and SARS-CoV-2 spike and authentic SARS-CoV-2 virus, including VOCs.

Prophylactic and therapeutic activity of selected VHH72-Fc constructs were assessed in SARS-CoV-2 challenged K18-hACE2 transgenic mice and hamsters. The sample size of the K18-hACE2 transgenic mice was estimated on the basis of experience with other respiratory viruses to give statistical power while minimizing animal use. Experimentalists involved in the mouse studies were not blinded, because objective measurements were used. To evaluate antiviral activity in the hamster model, we wanted to detect at least 1 log reduction of viral RNA in treated compared to untreated infected control animals. The group size was calculated on the basis of the independent *t* test with an effect size of 2.0 and a power of 80% (effect size = delta mean/SD = 1 log decrease in viral RNA/0.5 log), resulting in six animals per group. The sample sizes were maximized considering the limits in the biosafety level 3 (BSL3) housing capacity and the numbers of animals that can be handled under BSL3 conditions. All caretakers and technicians involved in the hamster studies were blinded to group allocation in the animal facility and to sample numbers for analysis [quantitative polymerase chain reaction (qPCR), titration, and histology].

### Molecular modeling of the VHH-72 (mutant) interaction with SARS-CoV-2 RBD

Molecular dynamics simulations were with model complexes of VHH72 [chain C from Protein Data Bank (PDB) entry 6WAQ] and variants, with the outward-positioned RBD from the cryo-EM structure PDB entry 6VSB of the SARS-CoV-2 prefusion spike glycoprotein (chain A, residues 335 to 528). The missing loops at residues 444 to 448, 455 to 490, and 501 to 502 in the cryo-EM RBD were reconstructed from the I-TASSER SARS-CoV-2 RBD model (*24*), and the missing residues were added by the use of Swiss-PDBViewer (*25*). Simulations of the VHH72(mutant)-RBD complex were with GROMACS version 2020.1 (*26*) using the Amber ff99SB-ILDN force field (*55*) and were run for 5 ns. After conversion of the trajectory to PDB format, snapshots were extracted every 0.5 ns and were submitted to the FastContact 2.0 server (*20*) for binding energy calculations.

### *E. coli* and *P. pastoris* strains

*Escherichia coli* MC1061 or DH5α were used for standard molecular biology manipulations. The *P. pastoris* (syn. *Komagataella phaffi*) NRRL-Y 11430 OCH1 function-inactivated strain used for VHH-Fc screening (*P. pastoris* OCH1) was obtained by CRISPR-Cas9 engineering (*56*). As reported before, the inactivation of the α-1,6-mannosyltransferase encoded by *OCH1* results in secretion of more homogenously glycosylated protein carrying mainly Man8 glycan structure (*57*).

### Modular generation of expression plasmids

The expression vectors for all the VHH72-XXX-hFc muteins were generated using an adapted version of the Yeast Modular Cloning toolkit on the basis of Golden Gate assembly (*58*). Briefly, coding sequences for the *Saccharomyces cerevisiae* α-mating factor minus EA-repeats (P3a_ScMF-EAEAdeleted), SARS-VHH72 mutants (P3b_SARS_VHH72-xxx), and hIgG1 hinge-hIgG1 Fc [P4a_(GGGGS)_x2_hIgG1.Hinge-hIgG1.Fc] were codon optimized for expression in *P. pastoris* using the GeneArt (Thermo Fisher Scientific) or Integrated DNA Technologies (IDT) proprietary algorithm and ordered as gBlocks at IDT (IDT BVBA, Leuven, Belgium). Each coding sequence was flanked by unique part-specific upstream and downstream Bsa I–generated overhangs. The gBlocks were inserted in a universal entry vector via BsmB I assembly, which resulted in different “part” plasmids, containing a chloramphenicol resistance cassette. Plasmids parts were assembled to form expression plasmids (pX-VHH72-xxx-hIgGhinge-hIgGFc) via a Golden Gate Bsa I assembly. Each expression plasmid consists of the assembly of nine parts: P1_ConLS, P2_pGAP, P3a_ScMF-EAEAdeleted, P3b_SARS_VHH72-xxx, P4a_(GGGGS)x2hIgG1.Hinge-hIgG1.Fc, P4b_AOX1tt, P5_ConR1, P6-7_Lox71-Zeo, and P8_AmpR-ColE1-Lox66. Selection was in LB supplemented with carbenicillin (50 μg/ml) and zeocin (50 μg/ml). All the part and expression plasmids were sequence verified. Transformations of linearized expression plasmids (*Avr*II) were performed using the lithium acetate electroporation protocol as described (*59*).

### Protein expression and purification

*P. pastoris* cultures were grown in liquid YPD (1% yeast extract, 2% peptone, and 2% d-glucose) or on solid YPD agar (1% yeast extract, 2% peptone, 2% d-glucose, and 2% agar) and selected with zeocin (100 μg/ml; InvivoGen). For small-scale expression screening, two to three single colonies of *P. pastoris* OCH1 transformed with pX-VHH72-xxx-hIgGhinge-hIgGFc were inoculated in 2 ml of BMDY [1% yeast extract, 2% peptone, 100 mM KH_2_PO_4_/K_2_HPO_4_, 1.34% YNB, and 2% d-glucose (pH 6)] or BMGY (same composition but with 1% glycerol replacing the 2% d-glucose) in a 24 deep-well block. After 50 hours of expression in a shaking incubator (28°C, 225 rpm), the medium was collected by centrifugation at 1500*g*, 4°C for 5 min. Protein expression was evaluated on Coomassie-stained SDS–polyacrylamide gel electrophoresis (SDS-PAGE) of crude supernatant. Crude supernatant was used immediately for analytics purposes (BLI and mass spectrometry, see below) or stored at −20°C.

VHH72-Fc constructs were produced in ExpiCHO cells (Thermo Fisher Scientific) by transient transfection of the respective expression plasmids. Cultures (25 ml) with 6 × 10^6^ cells/ml were grown at 37°C and 8% CO_2_ and transfected with 20 μg of pcDNA3.3-based plasmid DNA using ExpiFectamine CHO reagent. Twenty four hours after transfection, 150 μl of ExpiCHO enhancer and 4 ml of ExpiCHO feed were added to the cells, and the cells were further incubated at 32°C and 5% CO_2_. Transfected cells were fed a second time 5 days after transfection. As soon as cell viability had dropped below 75%, the culture medium was harvested. The VHH72-Fc constructs were purified from the clarified culture medium using a 5-ml MabSelect SuRe column (GE Healthcare). After a wash step with McIlvaine buffer (pH 7.2), bound proteins were eluted using McIlvaine buffer (pH 3). The eluted protein-containing fractions were neutralized with a saturated Na_3_PO_4_ buffer. These neutralized fractions were then pooled and loaded onto a HiPrep Desalting column for buffer exchange into storage buffer [25 mM l-histidine, 125 mM NaCl (pH 6)] or onto a HiLoad 16/600 Superdex 200-pg size exclusion column (GE Healthcare) calibrated with PBS or storage buffer. Where data are labeled as “XVR011,” protein material manufactured from a stable CHO cell line has been used in the experiments, of identical sequence as humVHH_S56A/LALA-Fc/Gen2 (which is the name that we use for the protein produced in ExpiCHO transient transfection).

Open reading frames (ORFs) corresponding to the light and heavy chains of the hIgG1 anti–SARS-CoV-2 antibody S309 were ordered synthetically at IDT. For both, an optimized Kozak sequence was added upstream of the start codon. The secretion signal of a *Mus musculus* Igκ chain was used to direct secretion of the S309 light chain and of a *M. musculus* IgG heavy chain to direct secretion of the S309 heavy chain. The C-terminal lysine residue of the S309 heavy chain was omitted. The synthetic DNA fragments were solubilized in ultraclean water at a concentration of 20 ng/μl, A-tailed using the NEBNext dA-tailing module (New England Biolabs), purified using CleanPCR magnetic beads (CleanNA), and inserted in pcDNA3.3-TOPO vector (Thermo Fisher Scientific). The ORF of positive clones was sequence verified, and pDNA of selected clones was prepared using the NucleoBond Xtra Midi Kit (Macherey-Nagel). Expression in ExpiCHO cells and purification of S309 was performed as described above for the VHH72-Fc constructs except that for S309 the heavy chain– and light chain–encoding plasmids were mixed in a ratio of 1:2. CB6 (heavy chain: GenBank, MT470197; light chain: GenBank, MT470196) was custom produced in a mammalian cell system by GenScript.

### Biolayer interferometry

The SARS-CoV-2 RBD binding kinetics of VHH72-hIgG1Fc affinity optimized variants in *P. pastoris* supernatant were assessed via BLI on an Octet RED96 system (FortéBio). Anti-mouse IgG Fc capture (AMC) biosensors (FortéBio) were soaked in kinetics buffer [10 mM Hepes (pH 7.5), 150 mM NaCl, bovine serum albumin (BSA; 1 mg/ml), 0.05% Tween 20, and 3 mM EDTA] for 20 min. Mouse IgG1 Fc-fused SARS-CoV-2 RBD (Sino Biological, 40592-V05H) at 5 to 15 μg/ml was immobilized on these AMC biosensors to a signal of 0.3 to 0.8 nm. Recombinant protein concentrations in crude cell supernatants of VHH72-hFc–expressing *P. pastoris* OCH1^−^ were estimated on the basis of band intensity on Coomassie-stained SDS-PAGE as compared to a purified VHH-hFc protein. Crude supernatants were diluted 20- to 100-fold in kinetics buffer to an approximate VHH72-hFc affinity mutant concentration of 5 to 10 nM. Association was measured for 180 s and dissociation for 480 s in similarly diluted supernatant of a nontransformed *P. pastoris* culture. Between analyses, biosensors were regenerated by three times 20-s exposure to regeneration buffer [10 mM glycine (pH 1.7)]. Using FortéBio Data Analysis 9.0 software, data were double-reference–subtracted, and the decrease of response signal during dissociation was determined. To measure the affinity of monovalent VHH72 variants for RBD, monomeric human Fc-fused SARS-CoV-2_RBD-SD1 (*27*) at 15 μg/ml was immobilized on anti-human IgG Fc capture (AHC) biosensors (FortéBio) to a signal of 0.35 to 0.5 nm. In an alternative setup, mouse IgG1 Fc-fused SARS-CoV-1 RBD (Sino Biological, 40150-V05H) or SARS-CoV-2 RBD (Sino Biological, 40592-V05H) at 15 μg/ml was immobilized on AMC biosensors (FortéBio). Association (120 s) and dissociation (480 s) of twofold dilution series starting from 200 nM VHH72 variant in kinetics buffer were measured. To assess full apparent binding kinetics of purified bivalent VHH72-hFc variants, bivalent mouse IgG1 Fc-fused SARS-CoV-2–RBD (Sino Biological, 40592-V05H) at 15 μg/ml was immobilized on AMC biosensors to a signal of 0.4 to 0.6 nm. Association (120 s) and dissociation (480 s) of twofold serial dilutions starting from 30 nM VHH72-hFc variants in kinetics buffer were measured. Between analyses of binding kinetics, AHC and AMC biosensors were regenerated by three times 20-s exposure to regeneration buffer [10 mM glycine (pH 1.7)]. Data were double-reference–subtracted and aligned to each other in Octet Data Analysis software v9.0 (FortéBio) on the basis of a baseline measurement of a nonrelevant VHH-IgG1 Fc fusion protein (for kinetics of VHH72-hFc variants) or kinetics buffer (for kinetics of monovalent VHHs). Association and dissociation of nonsaturated curves (both 1:1 kinetics of monovalent VHHs and 2:2 and 4:4 kinetics of bi- and tetravalent VHH-hFc variants) were fit in a global 1:1 model.

### Size exclusion chromatography–multiangle laser light scattering

To determine the molecular mass and aggregation behavior of VHH72-hFc variants, the protein was analyzed by size exclusion chromatography–multiangle laser light scattering (SEC-MALS). For each analysis, 100 μl of sample filtered through 0.1-μm Ultrafree-MC centrifugal filters (Merck Millipore) was injected onto a Superdex 200 Increase 10/300 GL Increase SEC column (GE Healthcare) equilibrated with sample buffer, coupled to an online ultraviolet (UV) detector (Shimadzu), a mini DAWN TREOS (Wyatt) MALS detector, and an Optilab T-rEX refractometer (Wyatt) at 298 K. The refractive index increment value (*dn*/*dc* value) at 298 K and 658 nm was calculated using SEDFIT v16.1 (*60*) and used for the determination of the protein concentration and molecular mass. Glycoprotein conjugate analysis was performed using a value of 0.140 ml/g for *dn/dc*_glycan_ [average carbohydrate *dn/dc* (*61*)] and a UV extinction coefficient of zero to account for the negligible absorbance of glycans at 280 nm. Data analysis was carried out using the ASTRA 7.3.2 software.

### SARS-CoV pseudovirus neutralization assay

To generate replication-deficient VSV pseudotyped viruses, HEK293T cells, transfected with SARS-CoV-1 S or SARS-CoV-2 S, were inoculated with a replication deficient VSV vector containing enhanced green fluorescent protein (GFP) and firefly luciferase expression cassettes (*62*, *63*). After a 1-hour incubation at 37°C, the inoculum was removed, and cells were washed with PBS and incubated in medium supplemented with an anti-VSV G mAb (American Type Culture Collection) for 16 hours. Pseudotyped particles were then harvested and clarified by centrifugation (*13*). For the VSV pseudotype neutralization experiments, the pseudoviruses were incubated for 30 min at 37°C with different dilutions of purified VHH or VHH-Fc fusions or with GFP-binding protein (GBP; a VHH specific for GFP). The incubated pseudoviruses were subsequently added to subconfluent monolayers of Vero E6 cells. Sixteen hours later, the cells were lysed using passive lysis buffer (Promega). The transduction efficiency was quantified by measuring the GFP fluorescence in the prepared cell lysates using a Tecan Infinite 200 Pro plate reader. GFP fluorescence was normalized using either the GFP fluorescence of noninfected cells and infected cells treated with PBS or the lowest and highest GFP fluorescence value of each dilution series. The IC_50_ was calculated by nonlinear regression curve fitting, log(inhibitor) versus response (four parameters).

### SARS-CoV-2 PRNT

The PRNT results shown in Fig. 2F, fig. S6E, and table S1 were performed with SARS-CoV-2 strain BetaCov/Belgium/GHB-03021/2020 [Environmental Performance Index (EPI) ISL 407976, 2020-02-03] used from passage P6 grown on Vero E6 cells (*14*). SARS-CoV-2 viruses belonging to the VoC UK and South African lineages B.1.1.7 (hCoV-19/Belgium/rega-12211513/2020; EPI_ISL_791333, 2020-12-21) and B.1.351 (hCoV-19/Belgium/rega-1920/2021; EPI_ISL_896474, 2021-01-11) were each isolated from nasopharyngeal swabs taken from travelers returning to Belgium in December 2020 and January 2021, respectively, and have recently been described (*64*). B.1.1.7 was from a healthy subject and B.1.351 from a patient with respiratory symptoms. Passage 2 B.1.1.7. and B.1.351 virus stocks were grown on Vero E6 cells and median tissue culture infectious doses (TCID_50_) defined by titration using the Spearman-Kärber method (*65*). Passage 0 and passage 2 B.1.1.7 and B.1.351 virus preparations had identical genome sequences.

Dose-dependent neutralization of distinct VHH-Fc constructs was assessed by mixing the VHH-Fc constructs at different concentrations (threefold serial dilutions starting from a concentration of 20 μg/ml), with 100 PFU of SARS-CoV-2 in Dulbecco’s Modified Eagle Medium (DMEM) supplemented with 2% fetal bovine serum (FBS), and by incubating the mixture at 37°C for 1 hour. VHH-Fc-virus complexes were then added to Vero E6 cell monolayers in 12-well plates and incubated at 37°C for 1 hour. Subsequently, the inoculum mixture was replaced with 0.8% (w/v) methylcellulose in DMEM supplemented with 2% FBS. After 3 days of incubation at 37°C, the overlays were removed, and the cells were fixed with 3.7% paraformaldehyde (PFA) and stained with 0.5% crystal violet. Half-maximum neutralization titers (PRNT_50_) were defined as the VHH-Fc concentration that resulted in a plaque reduction of 50% across two or three independent plates.

### Flow cytometric analysis of antibody binding to *Sarbecovirus* RBD displayed on the surface of *S. cerevisiae*

A pool of plasmids, on the basis of the pETcon yeast surface display expression vector, that encode the RBDs of a set of SARS-CoV2 homologs was generously provided by J. Bloom (*23*). This pool was transformed to *E. coli* TOP10 cells by electroporation at the 10-ng scale and plated onto low-salt LB agar plates supplemented with carbenicillin. Single clones were selected and grown in liquid low-salt LB supplemented with carbenicillin and miniprepped. Selected plasmids were Sanger sequenced with primers covering the entire RBD coding sequence, and the process was repeated until every desired RBD homolog had been picked up as a sequence-verified single clone. In addition, the CDS of the RBD of SARS-CoV2 was ordered as a yeast codon-optimized gBlock and cloned into the pETcon vector by Gibson assembly. The plasmid was transformed into *E. coli*, prepped, and sequence-verified as described above. DNA of the selected pETcon RBD plasmids was transformed to *S. cerevisiae* strain EBY100 according to the protocol by Gietz and Schiestl (*66*) and plated on yeast dropout medium (Synthetic Defined agar -trp -ura). Single clones were selected and verified by colony PCR for correct insert length. A single clone of each RBD homolog was selected and grown overnight in 10 ml of liquid repressive medium (SRaf -ura -trp) at 28°C. These precultures were then back-diluted to 50 ml of liquid inducing medium (SRaf/Gal -ura -trp) at an optical density at 600 nm (OD_600_) of 0.67/ml and grown for 16 hours before harvest. After washing in PBS, the cells were fixed in 1% PFA, washed twice with PBS, blocked with 1% BSA, and stained with dilution series of anti-RBD antibodies or palivizumab. Binding of the antibodies was detected using Alexa Fluor 633–conjugated anti-human IgG antibodies (Invitrogen). Expression of the surface-displayed myc-tagged RBDs was detected using a fluorescein isothiocyanate–conjugated chicken anti-myc antibody (Immunology Consultants Laboratory Inc.). After three washes with PBS containing 0.5% BSA, the cells were analyzed by flow cytometry using an BD LSRII flow cytometer (BD Biosciences). The binding curves were fitted using nonlinear regression (GraphPad Prism 8.0).

### Mouse challenge experiments

Transgenic (K18-hACE2)2Prlmn mice were originally purchased from the Jackson Laboratory. Locally bred hemizygous 10- to 14-week-old animals of both sexes were used for experiments. Infection experiments were performed in accordance with the guidelines of the Federation for Laboratory Animal Science Associations and the national animal welfare body. All experiments were in compliance with the German animal protection law and approved by the animal welfare committee of the Regierungspräsidium Freiburg (permit G-20/91).

WT-VHH/12GS-WT-Fc treatments were performed either by injecting about 500 μl of a solution of WT-VHH/12GS-WT-Fc (250 μg/ml) into the peritoneum of nonanesthetized mice to reach a dose of 5 mg/kg or by applying 40 μl of the WT-VHH/12GS-WT-Fc at 1 mg/ml to the nostrils of isoflurane-anesthetized mice. Infection of isoflurane-anesthetized mice with 8 × 10^3^ PFU of a clinical SARS-CoV-2 isolate (Muc-IMB-1/2020) was done by applying 40 μl of samples to the nostrils. Infected mice were monitored for weight loss and clinical signs of disease for 14 days. Mice were euthanized by cervical dislocation on day 3 after infection to determine infectious virus in lungs by plaque assay using Vero E6 cells. Infected mice were euthanized and scored dead if they lost 25% of their initial body weight or if showing severe signs of disease. All infection experiments were performed under BSL3 conditions.

### Hamster challenge experiments

The hamster infection model of SARS-CoV-2 performed in the J.N. laboratory, including the associated analytical procedures, has been described before (*67*). In brief, 6- to 8-week-old female Syrian hamsters (*Mesocricetus auratus*) were anesthetized with ketamine/xylazine/atropine and inoculated intranasally with 50 μl containing 2 × 10^6^ TCID_50_ SARS-CoV-2 BetaCov/Belgium/GHB-03021/2020. Animals were treated once by intraperitoneal injection, either 1 day before or 16 to 24 hours after SARS-CoV-2 challenge. Hamsters were monitored daily for appearance, behavior, and weight. At day 4 after infection, hamsters were euthanized by intraperitoneally injection of 500 μl of Dolethal [sodium pentobarbital (200 mg/ml); Vétoquinol, SA]. Lung, ileum, and stool were collected, and viral RNA and infectious virus were quantified by reverse transcription qPCR and end-point virus titration, respectively. Serum samples were collected at day 4 after infection for pharmacokinetics analysis. μCT data of in vivo hamster lungs were acquired using dedicated small animal μCT scanners, either using the X-cube (Molecubes, Ghent, Belgium) or the Skyscan 1278 (Bruker Belgium, Kontich, Belgium), as described earlier (*67*). Visualization and quantification of reconstructed μCT data were performed with DataViewer and CTan software (Bruker Belgium). Housing conditions and experimental procedures were approved by the ethics committee of animal experimentation of KU Leuven (license P065-2020).

The hamster model performed at Viroclinics was as follows. Male 14- to 15-week-old Syrian hamsters (*M. auratus*) weighing 106 to 158 g were obtained from Janvier. The animals were housed for 7 days in individually ventilated cages (two hamsters per cage) under BSL2 conditions before transfer to a BSL3 animal house. Six animals were used per group. Palivizumab (20 mg/kg), humVHH_S56A/LALAPG-Fc/Gen2 (20, 7, and 2 mg/kg), and (humVHH_S56A)_2_/LALAPG-Fc/Gen2 (20, 7, and 2 mg/kg) were administered by intraperitoneal injection in a volume of 1 ml per 100 g of hamster body mass 4 hours after the challenge. One group of hamsters received humVHH_S56A/LALAPG-Fc/Gen2 (20 mg/kg) by intraperitoneal injection 24 hours before challenge infection. All animals were infected intranasally with 10^4^ TCID_50_ of SARS-CoV-2 BetaCoV/Munich/BavPat1/2020 (passage 3, grown on Vero E6 cells) in a total volume of 0.1 ml. On day 4 after infection, all animals were euthanized by exsanguination under anesthesia.

Ethics approval for the hamster study performed at Viroclinics was registered under number: 277002015283-WP11. On day 2 after infection, throat swabs were collected in 1.5 ml of virus transport medium [Eagle’s Minimum Essential Medium containing BSA (fraction V), penicillin, streptomycin, amphothericin-B, l-glutamine, sodium bicarbonate, and Hepes], aliquoted, and stored. Upon necropsy, bronchoalveolar lavage (BAL) was performed on the left lung with 1 ml of PBS, and tissue samples were collected and stored in 10% formalin for histopathology and immunohistochemistry (left lung and left nasal turbinate) and frozen for virological analysis (right lung and right nasal turbinate). For virological analysis, tissue samples were weighed, homogenized in infection medium, and centrifuged briefly before titration. Serum samples on day 4 (about 500 μl per animal) after infection were collected during euthanization and immediately transferred to appropriate tubes containing a clot activator (Microvette 500 Z-Gel, Sarstedt, Germany). Throat swabs, BAL, and tissue homogenates were used to detect viral RNA. To this end, RNA was isolated (nucleic acid purification on the MagNA Pure 96; Roche Life Science) and reverse-transcribed, and Taqman PCR (on the 7500 Real-Time PCR system, Applied Biosystems) was performed using specific primers [E_Sarbeco: (forward) 5′-ACAGGTACGTTAATAGTTAATAGCGT-3′ and (reverse) 5′-ATATTGCAGCAGTACGCACACA-3′] and probe (E_Sarbeco_P1: 5′-ACACTAGCCATCCTTACTGCGCTTCG-3′) specific for betacoronavirus E gene. Quadruplicate 10-fold serial dilutions were used to determine the virus titers in confluent layers of Vero E6 cells. To this end, serial dilutions of the samples (throat swabs, BAL, and tissue homogenates) were made and incubated on Vero E6 monolayers for 1 hour at 37°. Vero E6 monolayers were then washed and incubated for 4 to 6 days at 37°, after which plates were stained and scored using the vitality marker WST8 (colorimetric readout). To this end, WST-8 stock solution was prepared and added to the plates. Per well, 20 μl of this solution (containing 4 μl of the ready-to-use WST-8 solution from the kit and 16 μl of infection medium, 1:5 dilution) was added and incubated 3 to 5 hours at room temperature. Subsequently, plates were measured for OD_450_ using a microplate reader, and visual results of the positive controls [cytopathic effect (cpe)] were used to set the limits of the WST-8 staining (OD value associated with cpe). Viral titers (TCID_50_/ml or TCID_50_/g) were calculated using the method of Spearman-Karber (*65*).

### Statistical analysis

Normal distribution of the data was tested by a Shapiro-Wilk test, and differences in variance among groups with normally distributed data were tested by an *F* test. For statistical analyses in which two groups with normally distributed data were compared, an unpaired Student’s *t* test was used. Welsh’s correction was applied for groups with significant difference in variance. When multiple groups with normally distributed data and no significant differences in variance were compared to the control group, a one-way analysis of variance (ANOVA) test and a Dunnet’s multiple comparisons test were used for post hoc analysis to correct for multiple comparisons. When multiple groups with not normally distributed data were compared to the control group, a Kruskal-Wallis test and a Dunn’s multiple comparisons test were used for post hoc analysis to correct for multiple comparisons. Differences in body weight between two groups were tested by two-way ANOVA with Sidak’s multiple comparison. Differences in survival were tested with a log-rank (Mantel-Cox) test. All statistical analysis was done using GraphPad Prism 9.10.

## Notes

View/request a protocol for this paper from *Bio-protocol*.
