# Supplementary material for: An affinity-enhanced, broadly neutralizing heavy chain–only antibody protects against SARS-CoV-2 infection in animal models
Source: Sci Transl Med. 2021 Nov 24;13(621):eabi7826. doi: 10.1126/scitranslmed.abi7826 (PMC9924070; doi:10.1126/scitranslmed.abi7826)
Supplement: Supplementary file 1 — Materials and Methods Figs. S1 to S16 Tables S1 to S8 [file scitranslmed.abi7826_sm.pdf]

Science Translational Medicine
AAAS

Supplementary Materials for

An affinity-enhanced, broadly neutralizing heavy chain–only antibody protects against SARS-CoV-2 infection in animal models

Bert Schepens *et al.*

Corresponding author: Nico Callewaert, nico.callewaert@vib-ugent.be; Xavier Saelens, xavier.saelens@vib-ugent.be

Sci. Transl. Med. **13**, eabi7826 (2021)
DOI: 10.1126/scitranslmed.abi7826

The PDF file includes:

Materials and Methods
Figs. S1 to S16
Tables S1 to S8

Other Supplementary Material for this manuscript includes the following:

Data files S1 and S2

Materials and Methods

Severe acute respiratory syndrome coronavirus 2 (SARS-CoV-2) spike sequence variant analysis. SARS-CoV-2 genome sequences originating from infected human hosts were downloaded from Global Initiative on Sharing All Influenza Data (GISAID) (N= 1,801,580 genomes available on June 2 2021). Genomes with invalid DNA character code were removed. Spike coding sequences were retrieved by aligning the genomes to the reference spike sequence annotated in NC_045512.2 (Wuhan-Hu-1 isolate, NCBI RefSeq). For this purpose, a pairwise alignment was performed using the R package Biostrings version 2.60.0, a fixed substitution matrix in the "overlap" mode with the following parameters according to Biostrings documentation: 1 and -3 for match and mismatch substitution scores; 5 as gap opening and 2 as gap extension penalties. Incomplete genomes without spike coding sequences, or generating very short or no alignment were removed. Coding sequences with frame-disturbing deletions were also excluded and the remaining open reading frames were in-silico translated using Biostrings option to solve "fuzzy" codons containing undetermined nucleotide(s). In the next step, predicted spike protein sequences with undetermined amino acids (denoted as X), derived from poor sequencing results (Ns) were removed. Further, full-length sequences with a single stop codon or lacking a stop signal (due to a possible C-terminal extension) were retained, and proteins with premature stop codon(s) were excluded.
The resulting 1,338,115 quality-controlled spike protein sequences were aligned using the ClustalOmega algorithm and R package multiple sequence alignment (msa) version 1.24.0 with default parameters and the BLOSUM65 substitution matrix. R packages seqinr 4.2-5 and Better ALignment CONsensus analYsis (BALCONY) 0.2.10 were used to calculate amino acid frequencies for all mutations occurring in the dataset at least once. Major and minor allele frequencies and counts were assigned. Effects of individual mutations on RBD yeast-surface display expression (a correlate of fold stability) were derived from Starr *et al* (*23*). Data collected for spike protein RBD (positions 333 – 518) was visualized using ggplot2 version 3.3.3.

Enzyme-linked immunosorbent assay. Wells of microtiter plates (type II, F96 Maxisorp, Nuc) were coated overnight at 4°C with 100 ng of recombinant SARS-CoV S-2P protein (with foldon), SARS-CoV-1 S-2P protein (with foldon), Fc-tagged SARS-CoV-2 RBD-SD1 or bovine serum albumin (BSA) (Sigma Aldrich). The coated plates were blocked with 5% milk powder in PBS. Dilution series of the VHHs were added to the wells. Binding was detected by incubating the plates sequentially with either horseradish peroxidase (HRP)-conjugated rabbit anti-camelid VHH antibody (Genscript, A01861-200), mouse anti-Histidine Tag antibody (MCA1396, Abd Serotec) followed by HRP-linked anti-mouse IgG (1/2000, NXA931, GE Healthcare) or Streptavidin-HRP (554066, BD Biosciences) or by HRP-linked rabbit anti-human IgG (A8792, Sigma Alrich). After washing, 50 µL of tetramethylbenzidine (TMB) substrate (BD OptETA) was added to the plates and the reaction was stopped by addition of 50 µL of 1 M H_2SO_4. The absorbance at 450 nM was measured with an iMark Microplate Absorbance Reader (Bio Rad). Curve fitting was performed using nonlinear regression (GraphPad Prism).

Polyethylene glycol (PEG) aggregation assay. Stock PEG 3350 (Merck, 202444) solutions (w/v) were prepared in PBS pH 7.4 or 50 mM histidine, 250 mM proline pH 5.5. A 1:1.1 serial titration was performed by an Assist plus liquid handling robot (Integra, 4505), to avoid liquid handling issues the stock concentration of PEG 3350 was capped at 40%. To minimize non-equilibrium precipitation, sample preparation consisted of mixing protein and PEG solutions at a 1:1 volume ratio. 35 µL of the PEG 3350 stock solutions was added to a 96-well v-bottom

polymerase chain reaction (PCR) plate (A1 to H1) by a liquid handling robot. 35 µL of a 2 mg/ml sample solution was added to the PEG stock solutions resulting in a 1 mg/ml test concentration. This solution was mixed by automated slow repeat pipetting, samples were then incubated at 20°C for 24 hours. The sample plate was subsequently centrifuged at 4000 x g for 1 hour at 20°C. 50 µL of supernatant was dispensed into a UV-Star, half area 96 well, µClear, microplate (Greiner, 675801). Protein concentrations were determined by UV spectrophotometry at 280 nm using a FLUOstar Omega multi-detection microplate reader (BMG LABTECH). The resulting values were plotted using GraphPad Prism and the PEG midpoint score was derived from the midpoint of the sigmoidal dose-response (variable slope) fit.

Hydrophobic interaction chromatography (HIC) assay. Apparent hydrophobicity was assessed using a hydrophobic interaction chromatography (HIC) assay employing a Dionex ProPac HIC-10 column, 100 mm×4.6 mm (Thermo Fisher 063655), containing a stationary phase consisting of a mixed population of ethyl and amide functional groups bonded to silica. All separations were carried out on an Agilent 1200 HPLC equipped with a fluorescence detector. The column temperature was maintained at 20°C throughout the run and the flow rate was 0.8 ml/minute. The mobile phases used for the HIC method were (A) 0.8 M ammonium sulfate and 50 mM phosphate pH 7.4, and (B) 50 mM phosphate pH 7.4. Following a 2 minute hold at 0% B, the column was loaded with 15 µl of sample at 2 mg/ml, and bound protein was eluted using a linear gradient from 0 to 100% B in 45 minutes and the column was washed with 100% B for 2 minutes and re-equilibrated in 0% B for 10 minutes prior to the next sample. The separation was monitored by absorbance at 280 nm.

Experimental isoelectric point (pI) measurement. An iCE3 whole-capillary imaged capillary isoelectric focusing (cIEF) system (ProteinSimple) was used to experimentally determine pI. Samples were prepared by mixing the following: 30 µl sample (from a 1 mg/ml stock in HPLC grade water), 35 µL of 1% methylcellulose solution (ProteinSimple, 101876), 4 µl pH 3-10 pharmalytes (ProteinSimple, 042-848), 0.5 µl of 4.65, 0.5 µl 9.77 synthetic pI markers (ProteinSimple, 102223 and 102219), and 12.5 µl of 8 M urea solution (Sigma Aldrich). HPLC grade water was used to make up the final volume to 100 µl. Samples were focused for 1 minute at 1.5 kV, followed by 5 minutes at 3 kV, 280 nm images of the capillary were taken using the Protein Simple software. The resulting electropherograms were analyzed using iCE3 software and pI values were assigned (linear relationship between the pI markers).

Mass spectrometry analysis of proteins. Intact VHH72-Fc protein (10 µg) was first reduced with tris(2-carboxyethyl)phosphine (TCEP; 10 mM) for 30 minutes at 37°C, after which the reduced protein was separated on an Ultimate 3000 HPLC system (Thermo Fisher Scientific) online connected to an LTQ Orbitrap XL mass spectrometer (Thermo Fischer Scientific). Briefly, approximately 8 µg of protein was injected on a Zorbax 300SB-C18 column (5 µm, 300Å, 1x250mm IDxL; Agilent Technologies) and separated using a 30 minute gradient from 5% to 80% solvent B at a flow rate of 100 µl/minute (solvent A: 0.1% formic acid and 0.05% trifluoroacetic acid in water; solvent B: 0.1% formic acid and 0.05% trifluoroacetic acid in acetonitrile). The column temperature was maintained at 60°C. Eluting proteins were directly sprayed in the mass spectrometer with an electrospray ionization (ESI) source using the following parameters: spray voltage of 4.2 kV, surface-induced dissociation of 30 V, capillary temperature of 325 °C, capillary voltage of 35 V and a sheath gas flow rate of 7 (arbitrary units). The mass spectrometer was operated in MS1 mode using the orbitrap analyzer at a resolution of 100,000 (at m/z 400) and a mass range of 600-4000 m/z, in profile mode. The resulting MS spectra were deconvoluted with the BioPharma Finder 3.0 software (Thermo Fisher Scientific)

using the Xtract deconvolution algorithm (isotopically resolved spectra). The deconvoluted spectra were manually annotated.

Peptide mapping by mass spectrometry. VHH72-Fc protein (15 µg) was diluted with 50 mM triethylammonium bicarbonate (pH 8.5) to a volume of 100 µl. First, protein disulfide bonds were reduced with dithiothreitol (DTT; 5 mM) for 30 minutes at 55°C and alkylated with iodoacetamide (IAA; 10 mM) for 15 minutes at room temperature in the dark. The protein was then digested with LysC endoproteinase (0.25 µg; NEB) for 4 hours at 37°C, followed by sequencing grade trypsin (0.3 µg; Promega) for 16 hours at 37°C. After digestion, trifluoroacetic acid was added to a final concentration of 1%. Prior to liquid chromatography-mass spectrometry (LC-MS) analysis, the samples were desalted using the Pierce C18 Spin Columns (Thermo Fisher Scientific). First, spin columns were activated with 400 µl 50% acetonitrile (2x) and equilibrated with 0.5% trifluoroacetic acid in 5% acetonitrile (2x), after which samples were slowly added on top of the C18 resin. The flow through of each sample was reapplied on the same spin column for 4 times to maximize peptide binding to the resin. After washing the resin with 200 µl of 0.5% trifluoroacetic acid in 5% acetonitrile (2x), peptides were eluted with two times 20 µl 70% acetonitrile. Desalted peptide samples were dried and resuspended in 50 µl 0.1% trifluoroacetic acid in 2% acetonitrile.
For the LC-MS/mass spectrometry (LC-MS/MS) analysis, 5 µl of the desalted peptide samples was injected on an in-house manufactured C18 column (ReprosilPur C18 (Dr.Maisch), 5 µm, 0.25x200mm IDxL) and separated using a 30 minute gradient from 0% to 70% solvent B at a flow rate of 3 µl/minute (solvent A: 0.1% formic acid and 0.05% trifluoroacetic acid in water; solvent B: 0.1% formic acid and 0.05% trifluoroacetic acid in 70% acetonitrile). The column temperature was maintained at 40°C. Eluting proteins were directly sprayed in the LTQ Orbitrap XL mass spectrometer with an ESI source using the following parameters: spray voltage of 4.2 kV, capillary temperature of 275 °C, capillary voltage of 35 V and a sheath gas flow rate of 5 (arbitrary units). The mass spectrometer was operated in data-dependent mode, automatically switching between MS survey scans and MS/MS fragmentation scans of the 3 most abundant ions in each MS scan. Each MS scan (m/z 250-3000) was followed by up to 3 MS/MS scans (isolation window of 3 Da, collision-induced dissociation (CID) collision energy of 35%, activation time of 30 ms) that fulfill predefined criteria (minimal signal of 5000 counts, exclusion of unassigned and single charged precursors). Precursor ions were excluded from MS/MS selection for 60 seconds after two selections within a 30 second time frame.
The resulting MS/MS spectra were analyzed with the BioPharma Finder 3.0 software (Thermo Fisher Scientific) and mapped onto the appropriate protein sequence. For peptide identification, the following parameters were used: maximum peptide mass of 7000 Da, mass accuracy of 5 ppm and a minimum confidence of 0.80. Cysteine carbamidomethylation was set as a fixed modification. Deamidation of asparagine and glutamine, pyroglutamate formation of N-terminal glutamine, glycation of lysine, and oxidation of methionine and tryptophan were set as variable modifications. The search for glycosylation modifications was enabled (CHO-specific). The maximum number of variable modifications per peptide was set at 3.

Physical and chemical stability testing. Dynamic light scattering was performed using the Uncle instrument (Unchained Labs). To evaluate the homogeneity and possible aggregate formation or WT-VHH/WT-Fc at 25°C, 10 µL of sample at 1.0, 20 or 30 mg/ml in 25 mM His and 125 mM NaCl, pH6.0 was added to the sample cuvette. Laser and attenuator controls were set at Auto and 10 acquisitions were run per data point with an acquisition time of 10 seconds for each. Intrinsic tryptophan-fluorescence was monitored upon temperature-induced protein unfolding in the Uncle instrument. Also, here, 10 µL of sample at 1 mg/ml was applied to the sample cuvette, and a linear temperature ramp was initiated from 25 to 95°C at a rate of

0.5°C/minute, with a pre-run incubation for 180 seconds. The barycentric mean (BCM) and static light scattering (SLS at 266 nm and 473 nm) signals were plotted against temperature in order to obtain melting temperatures (T_m) and aggregation onset temperatures (T_{agg}), respectively.
Accelerated temperature stress experiments were performed to accelerate the possible formation of soluble or insoluble protein aggregates as well as chemical isoforms, such as asparagine/glutamine deamidation, aspartate/glutamate isomerization, N-terminal pyro-glutamate and truncated species. Stability at elevated temperature was assessed by subjecting duplicate 200 µl 1 mg/ml protein samples (0.02% sodium azide added and 0.22 µm filtered) in 1.5 ml polypropylene Safe-Lock tubes (Eppendorf) to 10 days of storage at 40°C with shaking at 200 rpm in a thermomixer (Eppendorf Thermomixer Comfort 5355).

Flow cytometry analysis of binding to HEK293T or HEK293S cells expressing SARS-CoV-1 or SARS-CoV-2 spike protein. To investigate the binding of monomeric VHH72 and VHH72-Fc constructs to spike proteins on the surface of mammalian cells by flow cytometry, we used expression plasmids containing the coding sequence of the SARS-CoV-1 and SARS-CoV-2 spike proteins or of a SARS-CoV-1 spike protein in which the RBD was replaced by that of SARS-CoV-2 as described by Letko *et al.*[14]. The latter was used as a template to generate expression plasmids of the K417N, N439K, E484K, N501Y, and the combination of K417N + E484K + N501Y spike variants by QuickChange site-directed mutagenesis (Agilent) according to the manufacturer's instructions. HEK293S cells (Thermo Fisher Scientific) were cultured in FreeStyle293 expression medium (Life Technologies), cultured at 37°C with 8% CO_2 with shaking at 130 rpm. HEK293T cells (ATCC) and Vero E6 cells (ATCC) were cultured at 37°C in the presence of 5% CO_2 in Dulbecco's Modified Eagle Medium (DMEM) supplemented with 10% heat-inactivated fetal bovine serum (FBS), 1% penicillin, 1% streptomycin, 2 mM l-glutamine, non-essential amino acids (Invitrogen) and 1 mM sodium pyruvate. HEK293T cells were transfected with Fugene (Promega) and HEK293S cells with polyethyleneimine (Sigma Aldrich). Cells were transfected with spike expression plasmids each combined with a green fluorescent protein (GFP) expression plasmid. Two days after transfection, the cells were collected, washed once with PBS and fixed with 1% paraformaldehyde (PFA) for 30 minutes. Binding of human monoclonal antibodies palivizumab, CB6, and S309 and VHH72-Fc or variants thereof was detected with an Alexa Fluor (AF) 633 conjugated goat anti-human IgG antibody (Invitrogen). Binding of monomeric VHHs to SARS-CoV-1 or SARS-CoV-2 spike protein was detected with a mouse anti-HisTag antibody (AbD Serotec) and an AF647 conjugated donkey anti-mouse IgG antibody (Invitrogen). Following 3 washes with PBS containing 0.5% BSA, the cells were analyzed by flow cytometry using an BD LSRII flow cytometer (BD Biosciences). Binding was calculated as the mean AF633 fluorescence intensity (MFI) of GFP expressing cells (GFP^+) divided by the MFI of GFP negative cells (GFP^-). The binding curves were fitted using nonlinear regression (GraphPad Prism).

RBD competition assay on Vero E6 cells. SARS-CoV-2 RBD fused to murine IgG Fc (Sino Biological) at a final concentration of 0.4 µg/ml was incubated with a dilution series of monovalent VHH or VHH-Fc proteins and incubated at room temperature for 20 minutes followed by an additional 10 minutes incubation on ice. Vero E6 cells grown at sub-confluency in DMEM supplemented with 10% heat-inactivated FBS, 1% penicillin, 1% streptomycin, 2 mM l-glutamine, non-essential amino acids (Invitrogen) and 1 mM sodium pyruvate and were detached by cell dissociation buffer (Sigma Aldrich) and trypsin (Thermo Fisher Scientific) treatment. After washing once with PBS, the cells were blocked with 1% BSA in PBS on ice. All remaining steps were also performed on ice. The mixtures containing RBD and VHHs or VHH-Fc fusions were added to the cells and incubated for 1 hour. Subsequently, the cells were

washed 3 times with PBS containing 0.5% BSA and stained with an AF647 conjugated donkey anti-mouse IgG antibody (Invitrogen) for 1 hour. Following three additional washes with PBS containing 0.5% BSA, the cells were analyzed by flow cytometry using a BD LSRII flow cytometer (BD Biosciences).

Inhibition of ACE2 RBD interaction by AlphaLISA immunoassay. Dose-dependent inhibition of the interaction of SARS-CoV-2 RBD protein with the ACE2 receptor was assessed in a competition AlphaLISA (amplified luminescent proximity homogeneous assay). In brief, 2019-nCoV spike protein RBD that was biotinylated through an Avi-tag (AcroBiosystems, Cat nr. SPD-C82E9) was loaded on streptavidin coated Alpha Donor beads (Perkin Elmer, Cat nr. 6760002). Human ACE2-mFc protein (Sino Biological, Cat nr. 10108-H05H) was captured on anti-mouse IgG (Fc specific) acceptor beads (Perkin Elmer, Cat nr. AL105C). Serial dilutions of antibodies and VHH-Fc (final concentration ranging from 100nM to 0.001 nM) were made in assay buffer (PBS containing 0.5% BSA and 0.05% Tween-20), and mixed with biotinylated RBD protein (final concentration 1 nM) in white low binding 384well microtitre plates (F-bottom, Greiner Cat nr 781904). As isotype control antibody, palivizumab, was included. Subsequently, recombinant human ACE2-Fc (final concentration 0.2 nM) was added to the mixture. After an incubation for 1 hour at room temperature, donor and acceptor beads were added to a final concentration of 20 µg/ml for each in a final volume of 25 µl for an additional incubation of 1 hour at room temperature in the dark. Interaction between beads was assessed after illumination at 680 nm and reading at 615 nm on an Ensight instrument. Graph pad Prism was used for curve fitting and 50% inhibitory concentration (IC_{50}) determination of triplicate measurements.

Human membrane protein microarray assay. Retrogenix Ltd performed the specificity testing of XVR011 using a proprietary cell microarray technology (*37*). First, a pre-screen was undertaken to determine the amount of background binding of the test antibody to untransfected HEK293 cells, and to slides spotted with gelatin with or without SARS-CoV-2 spike protein. Slides were spotted with expression vectors encoding both ZsGreen1 and human CD20 or epidermal growth factor receptor (EGFR), and used to reverse-transfect HEK293 cells. After fixation, slides were spotted with 10% gelatin with or without SARS-CoV-2 full length (FL) Spike-6His (Peak Proteins Ltd; lot # 20200623-01-b1). XVR011 at concentrations of 1, 2.5, or 10 µg/mL, 1 µg/mL Rituximab biosimilar (positive control) or PBS, was then added to the slides. Binding to target-expressing cells and untransfected cells was assessed using an AF647 labelled anti-human IgG Fc detection antibody, followed by fluorescence imaging. Binding to soluble spotted SARS-CoV-2 FL Spike-6His was seen at all concentrations tested and for the full library screen, XVR011 was applied at a concentration of 10 µg/ml. For library screening, 5,475 expression vectors, encoding both ZsGreen1 and a full-length human plasma membrane protein or a cell surface-tethered human secreted protein, were individually arrayed in duplicate across 16 microarray slides (‘slide-sets’). Vectors encoding a further 371 human heterodimers were co-arrayed across a further microarray slide. An expression vector (pIRES-hEGFR-IRES-ZsGreen1) was spotted in quadruplicate on every slide, and was used to ensure that a minimal threshold of transfection efficiency had been achieved or exceeded on every slide. This minimal threshold had been defined previously. Human HEK293 cells were used for reverse transfection and expression. An additional slide was screened containing SARS-CoV-2 FL Spike-6His spotted in gelatin on top of fixed, untransfected HEK293 cells. Test antibody was added to each slide after cell fixation, giving a final concentration of 10 µg/ml. Detection of binding was performed by using the same fluorescent secondary antibody as used in the pre-screen. Two replicate slides were screened for each of the slide-sets. Fluorescent images were analyzed and quantitated (for transfection) using ImageQuant software. A protein ‘hit’ is defined as a

duplicate spot showing a raised signal compared to background. This is achieved by visual inspection using the images gridded on the ImageQuant software. Hits were classified as ‘strong, medium, weak or very weak’, depending on the intensity of the duplicate spots. In a subsequent Confirmation and Specificity screen, all 4 vector library hits, and 2 control receptors (CD20 and EGFR) were over-expressed in HEK293 cells and SARS-CoV-2 FL Spike-6His were spotted onto two slides in gelatin after cell fixation. Confirmatory slides were then probed with XVR011 at 10 µg/ml, Rituximab biosimilar at 1 µg/ml or PBS, followed by AF647 conjugated anti-human IgG Fc detection antibody.

Surface plasmon resonance analysis of Fcγ Receptor binding. Surface plasmon resonance (SPR) analyses of XVR011 binding to purified recombinant FcγRI, FcγRIIa (H167), FcγRIIa (R167), FcγRIIIa (V176), FcγRIIIa (F176), FcγRIIIb, and FcγRIIb were performed at Biaffin GmbH & Co KG on a Biacore T200 instrument (GE Healthcare). His-tagged recombinant human FcγRI, FcγRIIa (H167), FcγRIIa (R167), FcγRIIb, FcγRIIIa (V176), FcγRIIIa (F176), and FcγRIIIb were purchased from Acro Biosystems. Rituximab (Roch Diagnostics), a CD20-specific chimeric mouse-human monoclonal antibody with a human IgG1 Fc, was used as a control for Fcγ receptor binding. Anti-His monoclonal antibody was immobilized on an S sensor chip CM3 by covalent coupling and used to capture the His-tagged Fcγ receptors. Ligand capturing concentrations were as follows. FcγRI: 0.04 µg/ml and 0.08 µg/ml, FcγRIIA (H167): 0.05 µg/ml and 0.1 µg/ml, FcγRIIA (R167): 0.1 µg/ml and 0.2 µg/ml, FcγRIIB: 0.05 µg/ml and 0.1 µg/ml, FcγRIIIA (V176): 0.04 µg/ml and 0.1 µg/ml, FcγRIIIA (F176): 0.03 µg/ml and 0.12 µg/ml, and FcγRIIIB: 0.06 µg/ml and 0.15 µg/ml. Serially diluted XVR011 and Rituximab, diluted in 10 mM HEPES (pH 7.4), 150 mM NaCl, 3 mM EDTA, 0.05% Tween-20, were then injected on FcγR capture surfaces and analyzed in multi cycle kinetic mode (9-10 concentrations) at 50 µL/minute (association time: 1 minute; dissociation time: 5 to 10 minutes). The sensor chips were regenerated with 10 mM of Glycine pH 1.5 (two consecutive 30 second injections) followed by 3 M of guanidine hydrochloride (two consecutive 30 second injections). Data processing (double referencing including blank run subtraction) and evaluation was performed by steady state analysis using Biacore T200 Evaluation Software version 3.1. Statistical evaluation (mean, standard deviation [SD]) was conducted using Microsoft Excel.

Quantification of RBD-binding VHH-Fcs in serum samples of challenged hamsters. Concentrations of XVR011 and preleads in hamster serum samples were quantified in a competition AlphaLISA as described in the main article text. This assay detects the inhibition of the interaction of SARS-CoV-2 RBD protein with monovalent humVHH72_S56A. This homogeneous assay without wash steps in a closed system is considered advantageous for testing samples from virus-challenged animals. In standards, spiked controls and diluted samples were mixed with 3 nM VHH72-h1 (S56A)-Flag3-His3 and 2.5 nM biotinylated SARS-CoV-2 RBD protein in white low binding 384-well microtiter plates (F-bottom, Greiner Cat nr 781904). After an incubation for 1 hour at room temperature, donor and acceptor beads were added to a final concentration of 20 µg/ml for an additional incubation of 1 hour at room temperature in the dark. Interaction between beads was assessed after illumination at 680 nm and reading at 615 nm on an Ensight instrument. Standard curves were generated by 1.7-fold serial dilutions of the respective compound in pooled hamster serum diluted in alphascreen assay buffer (PBS containing 0.5% BSA and 0.05% Tween-20). Concentrations were back-calculated by 4 parameter logistic (4PL) interpolation using GraphPad Prism.

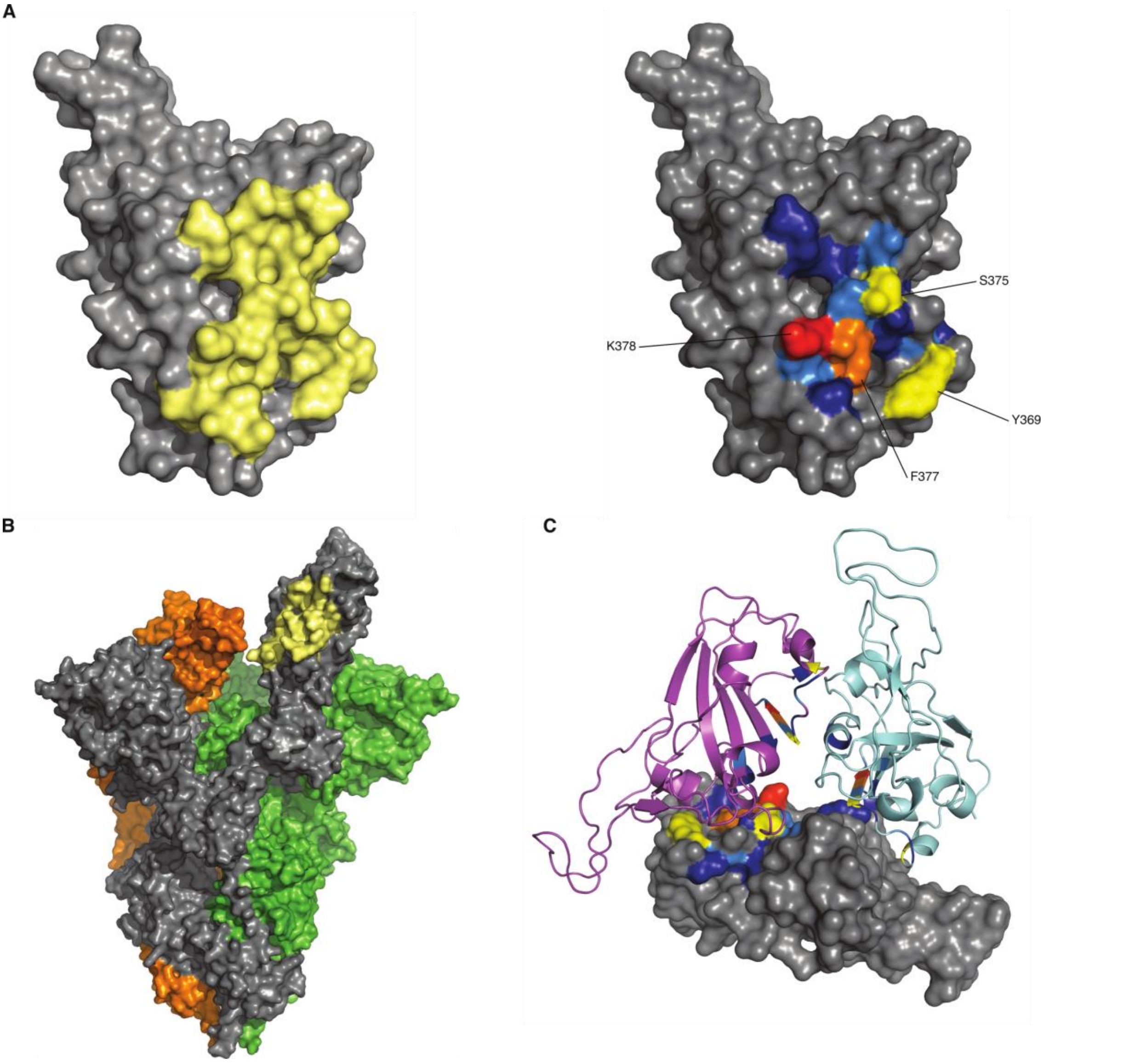

Fig. S1. The VHH72 contact region in SARS-CoV-2 RBD. (**A**) Left: RBD as surface-view with the VHH72 contact region indicated in yellow, for which PDB Protein Interfaces, Surfaces And Assemblies (PDBePISA) predicts residues 368-379, 381-385, 404, 405, 407, 408, 435-437, 503, 504 and 508. Right: relative contribution of the contact residues based on electrostatic plus desolvation free energy (kcal/mol) per residue calculated by FastContact. F377 and K378 represent a prominent hot-spot for VHH72 binding, as does Y369, the preferential orientation of which appears different between SARS-CoV-1 and SARS-CoV-2. Red: -9.8 (K378); orange: -4.3 (F377); yellow: -2.21 (S375) and -2.01 (Y369); light blue: -2.0 to -1.0 (A372, T376, C379 and Y508); dark blue: -1.0 to -0.5 (S371, F374, P384, V407, R408, T436). (**B**) Location of the contact region of VHH72 (light yellow) on a SARS-CoV-2 pre-fusion spike protein with one RBD in 'up' position (protomer in gray). Shown with a surface view of the spike protein from pdb-entry 7DD8, originally complexed with a Fab of the mouse monoclonal antibody 3C1. (**C**) The contact region of VHH72 is occluded in the RBD-closed state of SARS-CoV-2 spike pre-fusion protein. Shown is an apex-view of intact wild-type SARS-CoV-2 pre-fusion closed state spike trimer pdb-entry 6XR8, showing only the three RBDs. Chain A, gray-surface; chain B, cyan-cartoon; chain C, magenta-cartoon. The residues at the VHH72 binding contact region are shown with the same FastContact contribution colors as above.

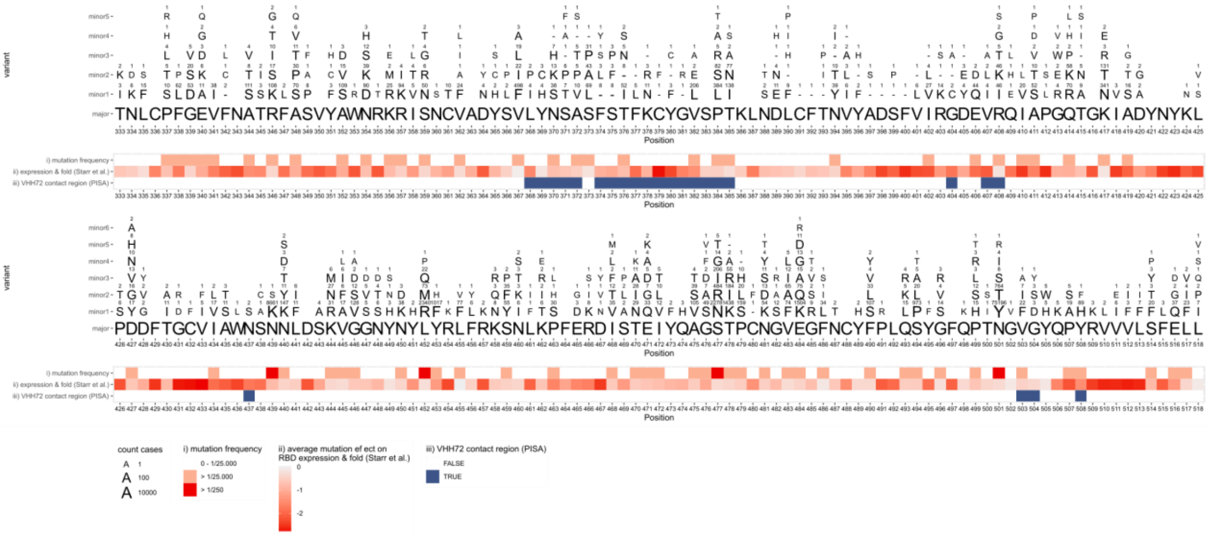

Fig. S2. The contact region of VHH72 is highly conserved across circulating SARS-CoV-2 viruses. The amino acid sequence of SARS-CoV-2 RBD (spike protein amino acid positions 333-518 of Wuhan-Hu-1 isolate) is shown with all missense mutations detected at least once in 1,338,115 SARS-CoV-2 genomes analyzed, depicted above each residue. Variants are ordered vertically at each position, according to frequency represented by data point size and the number of observed cases. i) Mutation frequency (white: low mutation frequency less than 1 per 25.000; pink: intermediate mutation frequency; red: high mutation frequency greater than 1 per 250), ii) average mutation effect on the RBD yeast surface display expression as a proxy for fold stability, according to Starr *et al.*[23] (white: no effect; red: strong negative effect), iii) the VHH72 contact region according to PISA calculations.

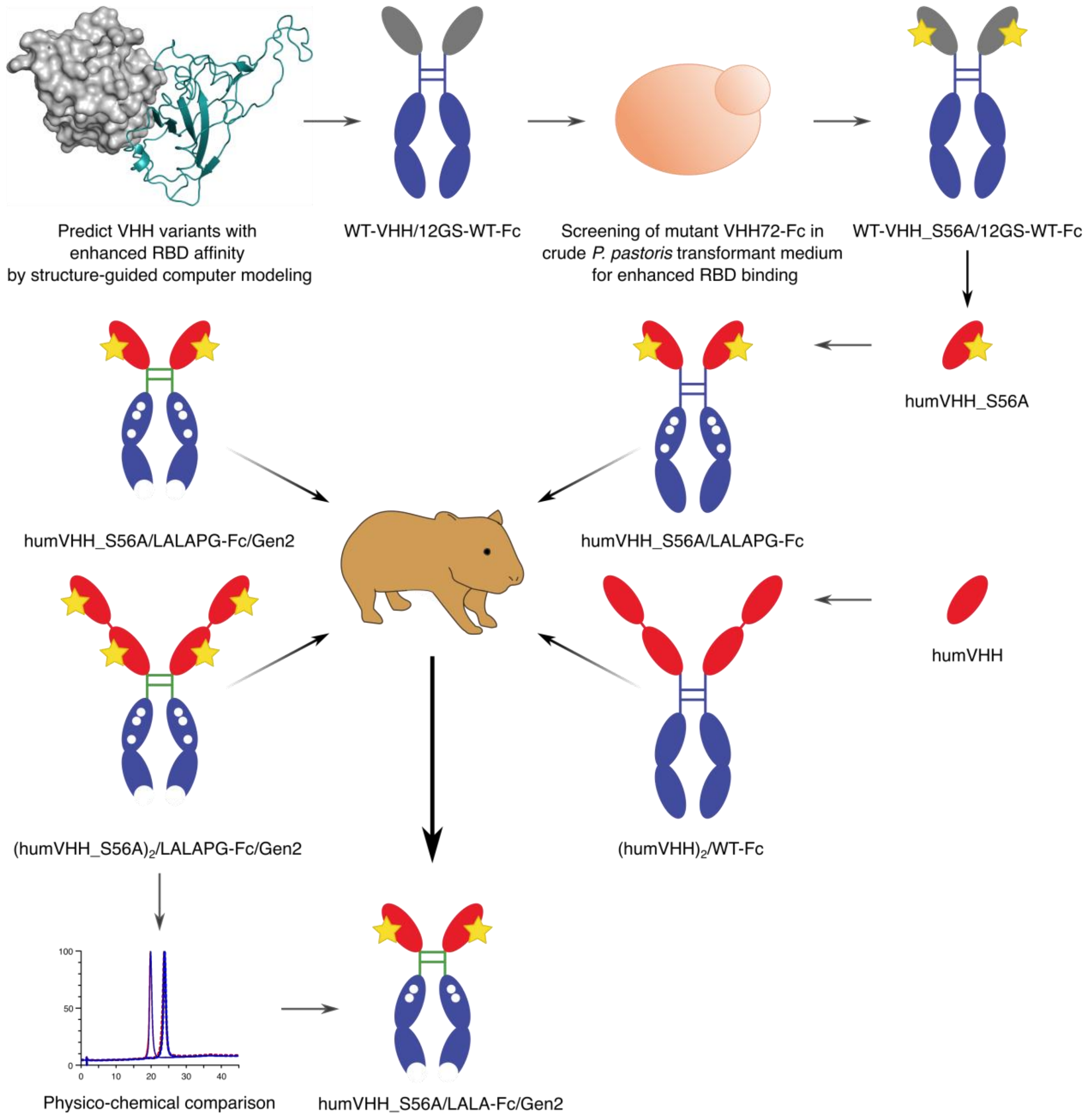

Fig. S3. Schematic overview of the VHH72-Fc development trajectory that resulted in XVR011. Structure-based computer modelling was applied to predict mutations in VHH72 that could result in enhanced affinity for the RBD of SARS-CoV-2. Mutants were generated in the VHH72-Fc context and expressed in *Pichia pastoris*. VHH72-Fc muteins in the crude yeast medium were screened for enhanced binding to immobilized recombinant SARS-CoV-2 RBD, which resulted in the identification of VHH72_S56A. The monomeric VHH72_S56A was subsequently humanized, the N-terminal glutamine replaced by glutamate resulting in humVHH_S56A. The humanized VHH72_S56A was then fused by a short GS linker to the hinge and Fc domain of a human IgG1 Fc, with or without LALAPG mutations. A tetravalent construct was generated in parallel based on humanized VHH72 (humVHH) to increase the RBD binding affinity. In vivo protection in the hamster model revealed comparable protection by the humanized S56A bivalent VHH-Fc and the tetravalent (humVHH)2-Fc. Next, the N-terminal glutamate was replaced by an aspartate, the hinge was further truncated through removal of EPKSC, the C-terminal lysine was removed, and the tetravalent construct was

combined with the S56A mutation in the VHH. These generation 2 Fc (Gen2) nanobody-Fc constructs were again assessed in the hamster challenge model. Finally, the somewhat less favorable physico-chemical properties of the tetravalent constructs and the decision to proceed with the LALA Fc modification led to the construction of humVHH_S56A/LALA-Fc/Gen2, the cGMP-produced counterpart of which was named XVR011.

A

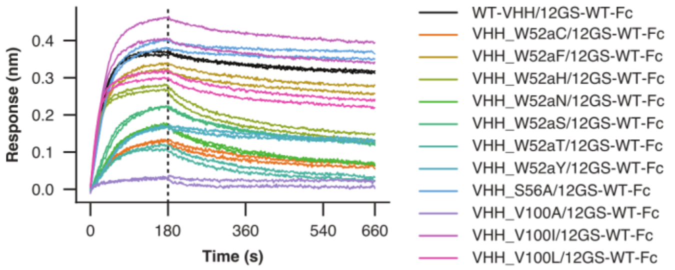

B

Variant	Affinity mutation			n	% signal decrease during 480s dissociation
	position	original	mutation		averaged after double reference subtraction
WT-VHH/12GS-WT-Fc	-	-	-	24	13.0
VHH_W52aC/12GS-WT-Fc	52a	W	C	2	52.8
VHH_W52aF/12GS-WT-Fc	52a	W	F	2	19.1
VHH_W52aH/12GS-WT-Fc	52a	W	H	2	49.1
VHH_W52aN/12GS-WT-Fc	52a	W	N	2	60.5
VHH_W52aS/12GS-WT-Fc	52a	W	S	2	45.0
VHH_W52aT/12GS-WT-Fc	52a	W	T	2	75.5
VHH_W52aY/12GS-WT-Fc	52a	W	Y	2	23.2
VHH_S56A/12GS-WT-Fc	56	S	A	2	7.8
VHH_V100A/12GS-WT-Fc	100	V	A	2	51.9
VHH_V100I/12GS-WT-Fc	100	V	I	2	15.3
VHH_V100L/12GS-WT-Fc	100	V	L	2	25.7

C

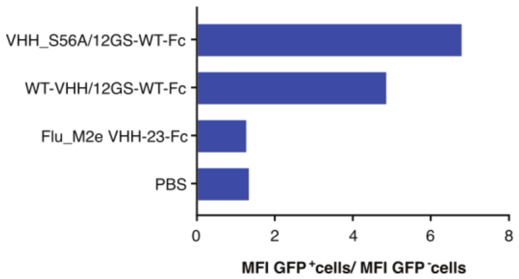

Fig. S4. Selection of VHH72_S56A-Fc. (**A**) Association (180 seconds) and dissociation (480 seconds) of duplicate WT-VHH/12GS-WT-Fc and VHH-mutants in diluted crude *Pichia pastoris* supernatant on SARS-CoV-2 RBD-mFc coated biosensors is shown. (**B**) Signal decrease during the dissociation phase is shown for the indicated VHH/12GS-WT-Fc variants expressed in crude *Pichia pastoris* supernatant. (**C**) The S56A substitution increases the binding of VHH/12GS-WT-Fc for the SARS-CoV-2 spike protein expressed on the surface of mammalian cells. HEK293T cells were cotransfected with a GFP and a SARS-CoV-2 spike expression vector and then stained with two-fold diluted crude *Pichia pastoris* supernatant expressing the indicated VHH-Fc proteins and analyzed by flow cytometry. Flu_M2e VHH-23-Fc is directed against the influenza A virus matrix 2 protein and was included as a negative control. The bars in the graph represent the ratio of the mean fluorescence intensity (MFI) of the GFP$^+$ over the MFI of the GFP$^-$ cells.

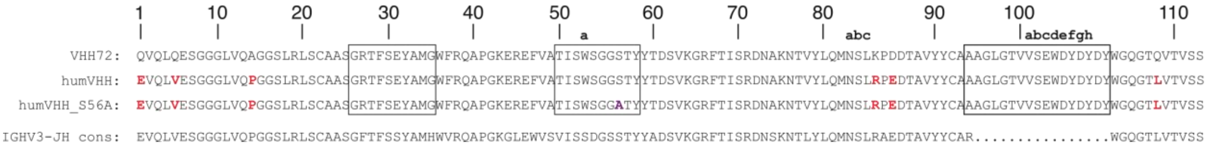

Fig. S5. Humanization of VHH72. Amino acid residue sequences of VHH72, humVHH, humVHH_S56A, and the human IGHV3-JH consensus sequence are shown. Boxed amino acids indicate CDR1, -2, and -3 in VHH72, humVHH, and humVHH_S56A. Amino acid residues in red represent the mutations in the framework regions that were introduced to generate the humVHH variant. Substitution S56A is marked in purple. In the generation 2 Fc (Gen2) nanobody-Fc constructs, the N-terminal residue was replaced by an aspartic acid residue to avoid all possibility for N-terminal heterogeneity.

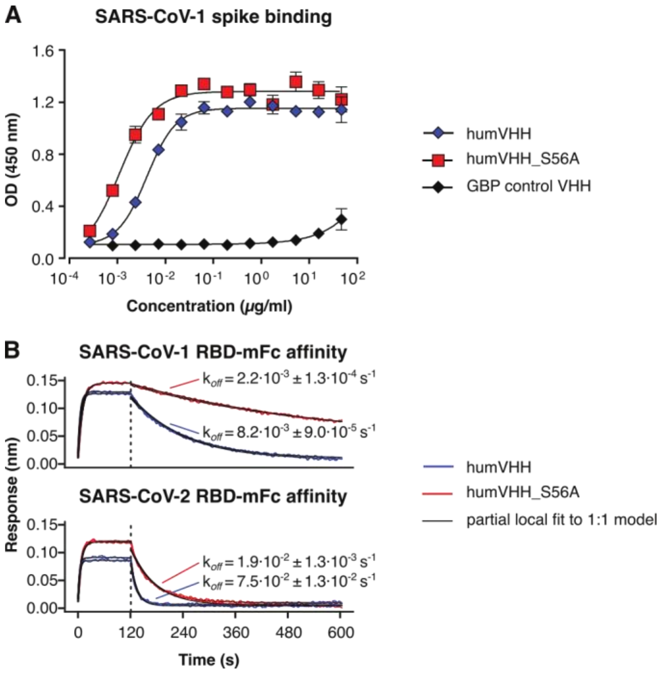

Fig. S6. HumVHH_S56A has increased affinity for SARS-CoV-1 spike protein and RBD. (**A**) Binding to SARS-CoV-1 spike protein was determined by enzyme-linked immunosorbent assay (ELISA). Data points represent mean ± SEM; N=3. (**B**) BLI sensorgram are shown for humVHH and humVHH_S56A (each 200 nM) binding to immobilized RBD from SARS-CoV-1 and SARS-CoV-2 fused to mouse Fc. Red (humVHH_S56A) and blue (humVHH) curves represent double reference-subtracted data and a partial local fit of the data to a 1:1 binding curve is colored black. Dissociation constants indicated (k_{off}) are mean and standard deviation of duplicate measures. GBP indicates GFP-binding protein (a VHH directed against GFP).

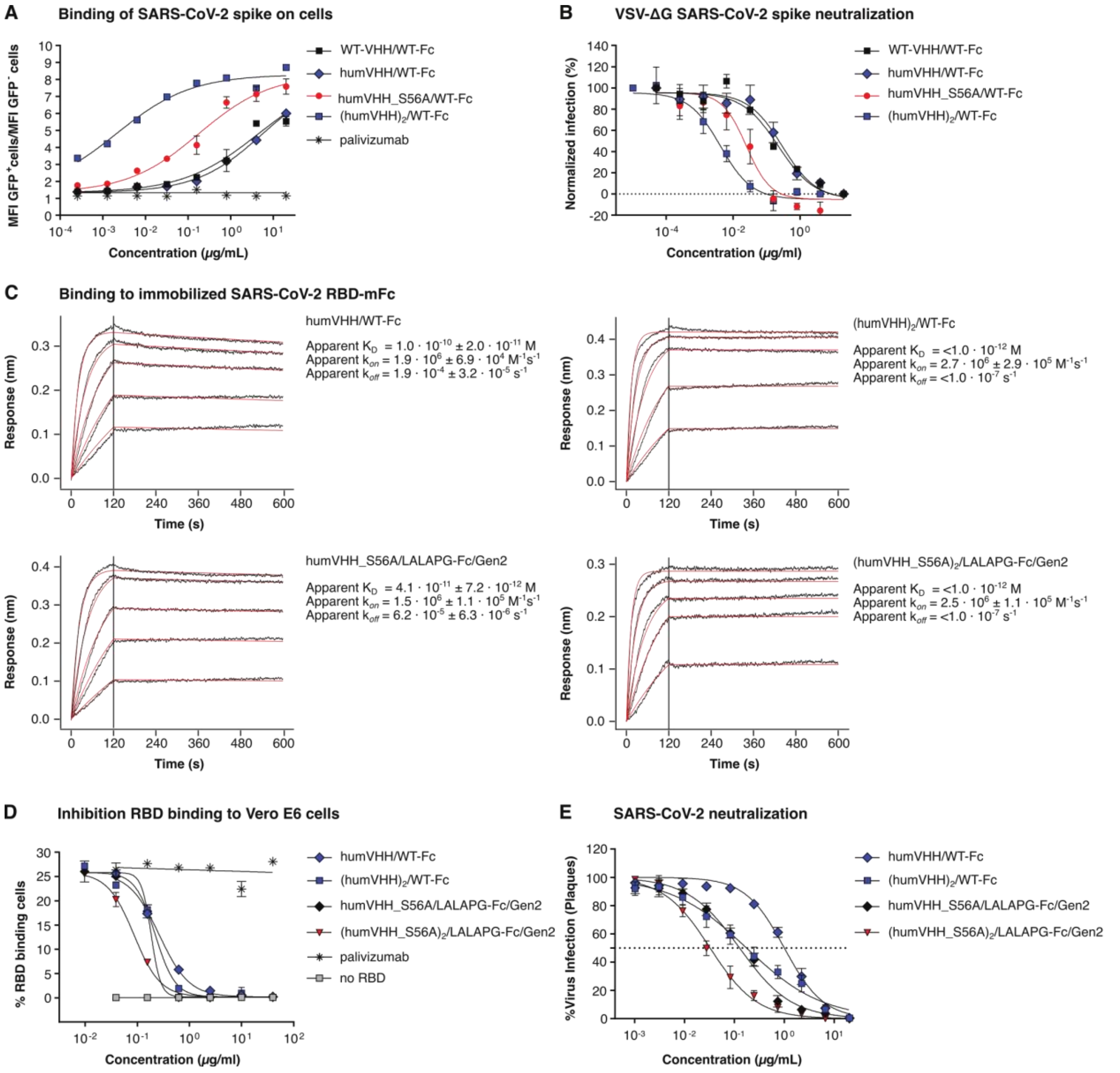

Fig. S7. Tetravalent VHH72-Fc has increased affinity for SARS-CoV-2 RBD. (**A**) Binding of the indicated VHH72-Fc constructs to mammalian cell surface expressed SARS-CoV-2 spike was determined by flow cytometry. The graph shows the mean (N=2, N=1 for (humVHH)$_2$/WT-Fc) ratio of the MFI of transfected (GFP$^+$) cells over the MFI of non-transfected (GFP$^-$) cells. Data are the same as in Fig. 3C except that tetravalent (humVHH)$_2$/WT-Fc is included here. (**B**) VSV SARS-CoV-2 spike pseudotype virus neutralization was evaluated with the indicated VHH72-Fc constructs. The graph shows the mean (N=3 ± SEM) GFP fluorescence normalized to the lowest and highest value of each dilution series. Data for WT-VHH/WT-Fc, humVHH/WT-Fc, and humVHH_S56A/WT-Fc are the same as shown in Fig. 3G. (**C**) Apparent binding affinity of bivalent (humVHH/WT-Fc and humVHH_S56A/LALAPG-Fc/Gen2) versus tetravalent (humVHH)$_2$/WT-Fc and (humVHH_S56A)$_2$/LALAPG-Fc/Gen2) VHH72-Fc variants (two-fold dilution series starting at 30 nM) to immobilized mouse Fc fused SARS-CoV-2 RBD (RBD-mFc). Black lines represent double reference-subtracted data and the fit of the 2:2 or 4:4 data to a 1:1 binding curve is colored red. A representative experiment of two distinct BLI analyses is shown. Apparent kinetics parameters are averages of duplicate experiments. (**D**) Dose-dependent inhibition of SARS-CoV-2 RBD binding to the surface of Vero E6 cells in the presence of the indicated VHH72-Fc constructs was determined by flow

cytometry. The graph shows the mean ± SD (N=2) percentage of cells that bind RBD. Data for humVHH/WT-Fc, PBS and no RBD are the same as in Fig. 3E. (**E**) SARS-CoV-2 plaque reduction neutralization assay with three-fold serial dilutions of the indicated VHH-Fc fusion constructs. Thirty-six hours after infection, the cells fixed and stained for analysis. Data points in the graph represent the relative mean ± SEM (N=3) number of plaques and are from two replicates of one experiment. Data for humVHH/WT-Fc and $(humVHH)_2$/WT-Fc are the same as in Fig. 3F.

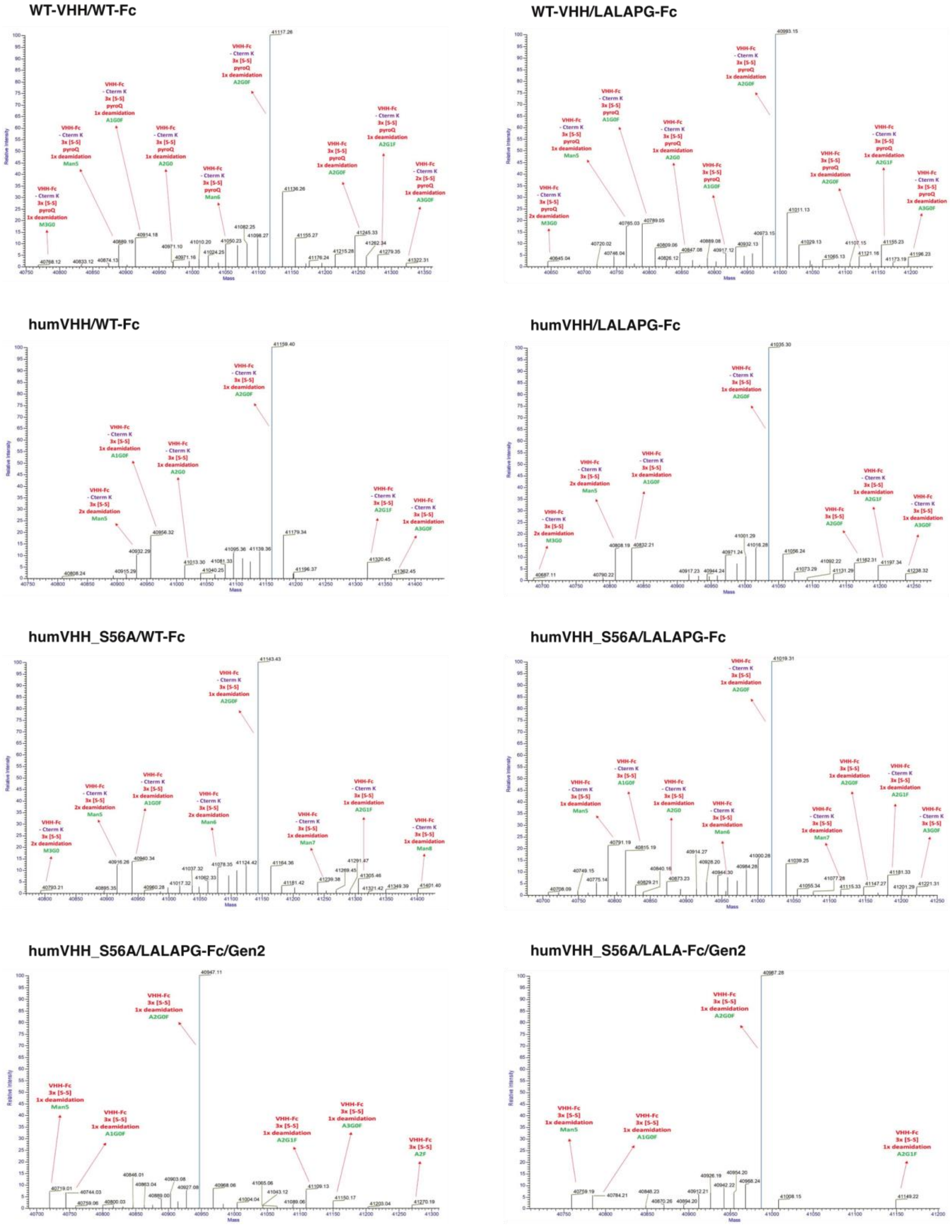

Fig. S8. Intact protein mass spectrometry analysis of different VHH72-Fc constructs. Proteins were first reduced, then separated with reversed phase liquid chromatography, and finally analyzed with an Orbitrap mass spectrometer. The panels depict the deconvoluted spectra of the different protein constructs.

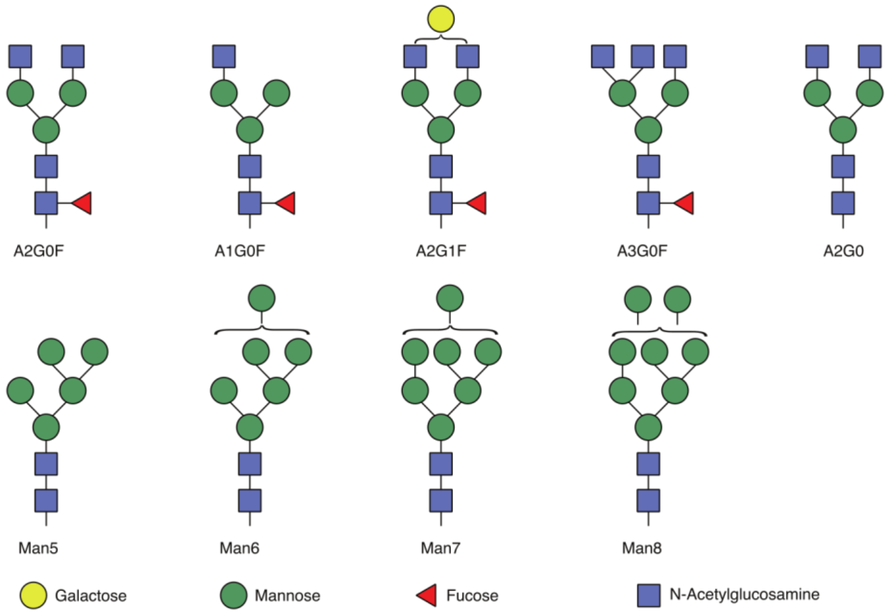

Fig. S9. Glycan codes and corresponding glycan composition of the detected N-glycans.

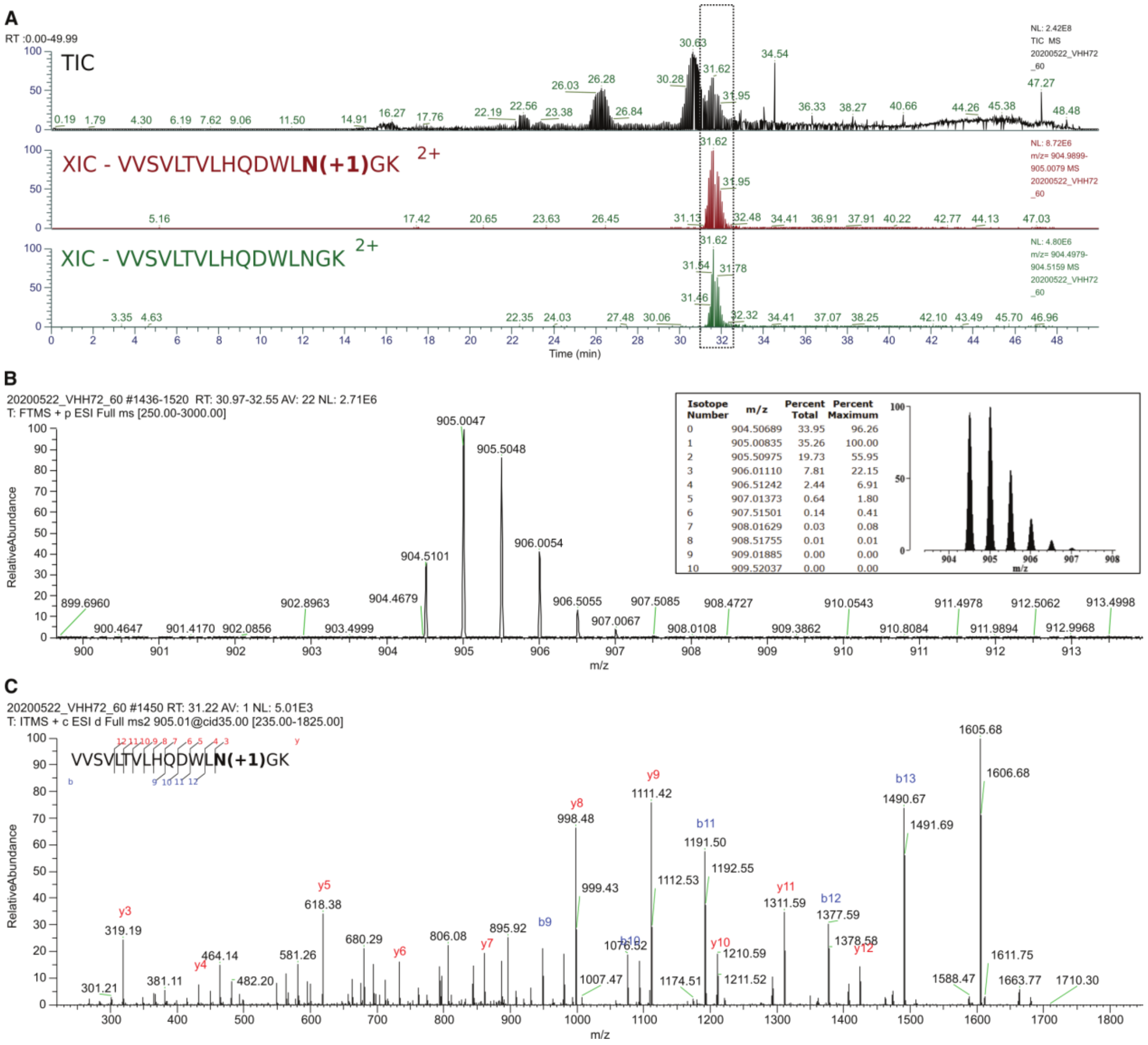

Fig. S10: N227 deamidation analysis of humVHH_S56A/WT-FC. (**A**) The upper chromatogram shows the total ion current (TIC) of the digested humVHH_S56A/WT-Fc. The two panels below show the extracted ion chromatograms (XIC) of the tryptic peptide with deamidated N227 (middle) or non-modified N227 (bottom). XICs have a mass tolerance of 10 ppm. (**B**) Summation of the time window indicated by the dotted line in panel (A), zoomed in on the double charged peptide containing N227. The expected isotopic envelope of the non-modified peptide is shown on the right, generated by MS-Isotope in ProteinProspector 6.2.3. The distorted experimental isotope envelope is indicative for deamidation. (**C**) CID MS2 spectrum of 905.01 Da precursor peak. Fragment ions match with the theoretical y and b ion series of the N227 deamidated peptide.

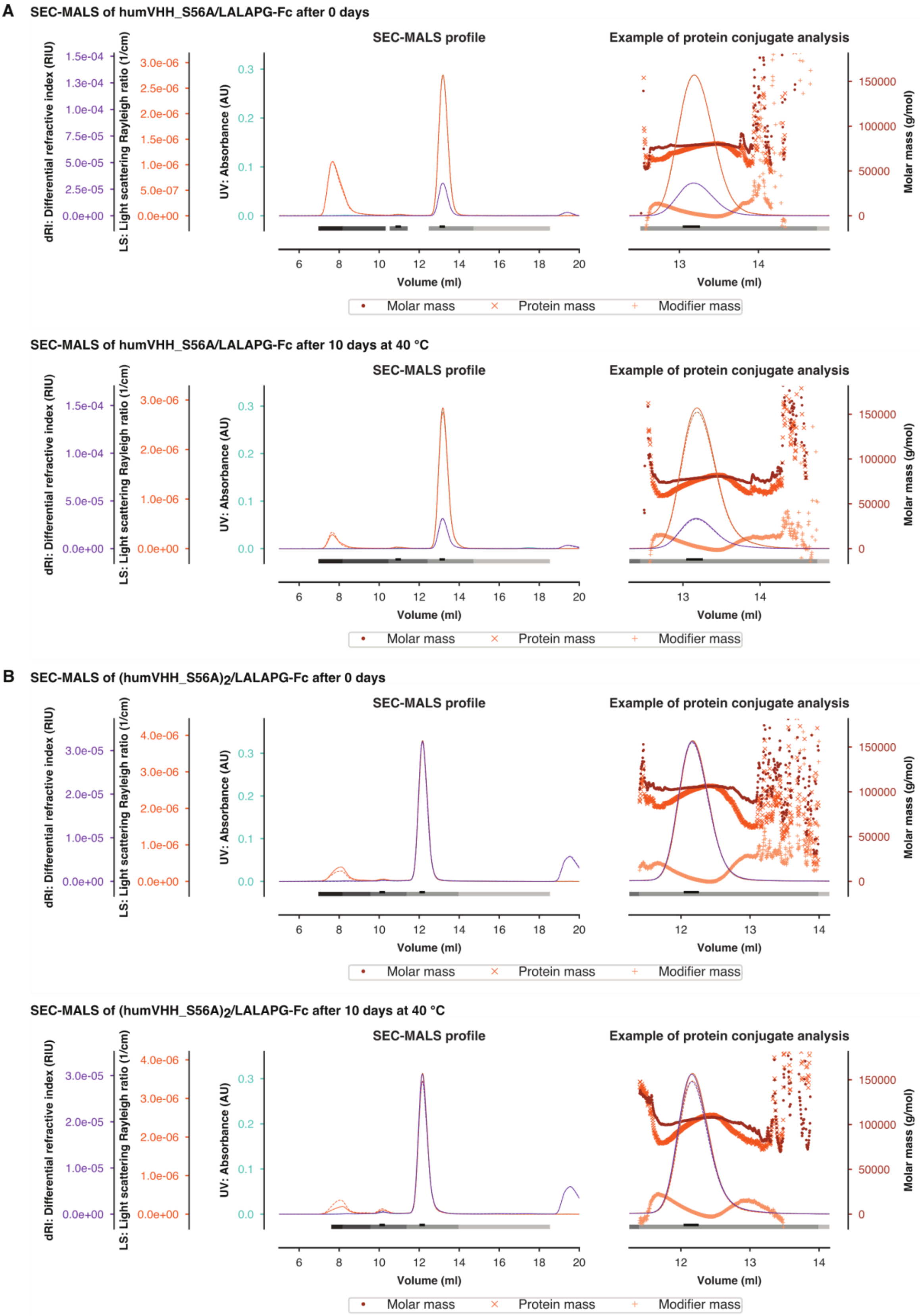
A
SEC-MALS of humVHH_S56A/LALAPG-Fc after 0 days
SEC-MALS of humVHH_S56A/LALAPG-Fc after 10 days at 40 °C
B
SEC-MALS of (humVHH_S56A)2/LALAPG-Fc after 0 days
SEC-MALS of (humVHH_S56A)2/LALAPG-Fc after 10 days at 40 °C
SEC-MALS profile
Example of protein conjugate analysis
dRI: Differential refractive index (RIU)
LS: Light scattering Rayleigh ratio (1/cm)
UV: Absorbance (AU)
Molar mass (g/mol)
Volume (ml)
Molar mass
Protein mass
Modifier mass

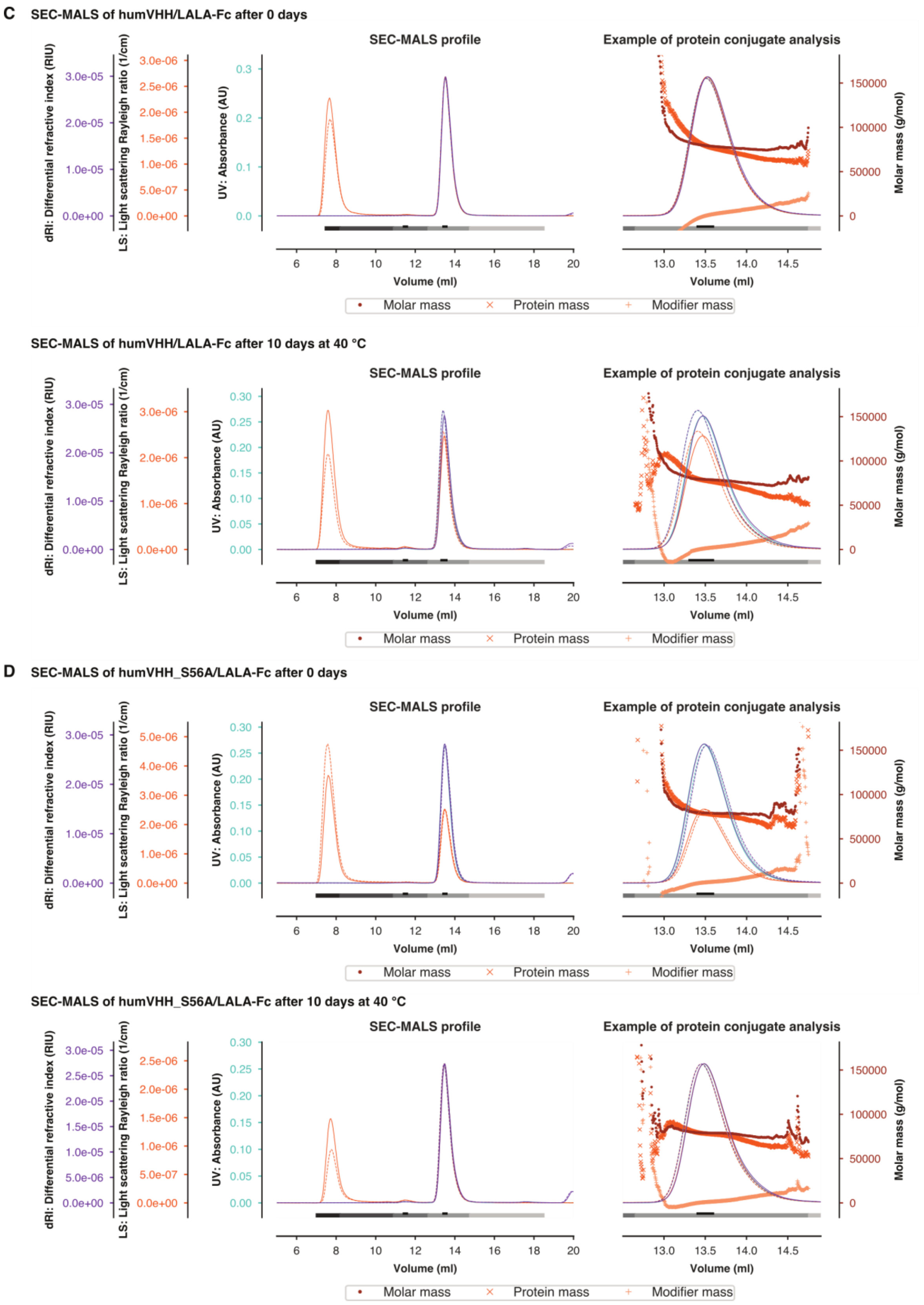

Fig. S11. VHH72-Fc constructs are stable in accelerated stress tests. Ten day-storage at 40°C causes no major changes in SEC-MALS profiles of 1 mg/ml of (**A**) humVHH_S56A/LALAPG-Fc/Gen2, (**B**) (humVHH_S56A)2/LALAPG-Fc/Gen2, (**C**)

humVHH/LALA-Fc/Gen2, and (**D**) humVHH_S56A /LALA-Fc/Gen2 in PBS. Complete SEC-MALS profiles of independent duplicates (solid and dashed lines). Peaks for quantitative analysis are indicated in grey below the spectra, peak apex indicated in black for qualitative analysis (results in table S7). The protein conjugate analysis was performed based on the differential extinction coefficients and refractive index values of proteins versus conjugated glycan modifiers.

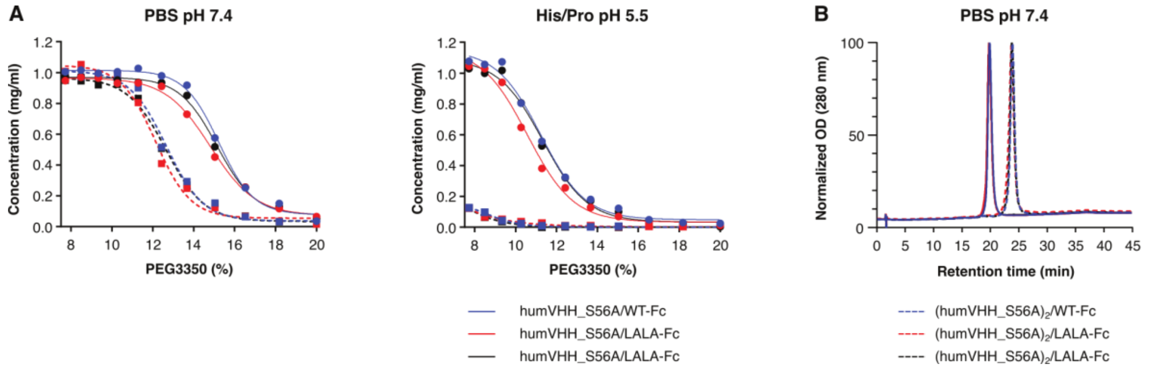

Fig. S12. Tetravalent (humVHH_S56A)₂/Fc constructs show reduced solubility and greater apparent hydrophobicity compared to the bivalent constructs. (**A**) A PEG aggregation assay was used to assess solubility of bivalent versus tetravalent VHH-Fc constructs in PBS pH 7.4 (left) or 50 mM histidine, 250 mM proline buffer pH 5.5 (right). (**B**) Hydrophobic interaction chromatography was used to compare bivalent versus tetravalent VHH-Fc constructs in 50 mM phosphate and 0.8M ammonium sulfate pH 7.4, eluted in a 45 min linear gradient to zero ammonium sulfate.

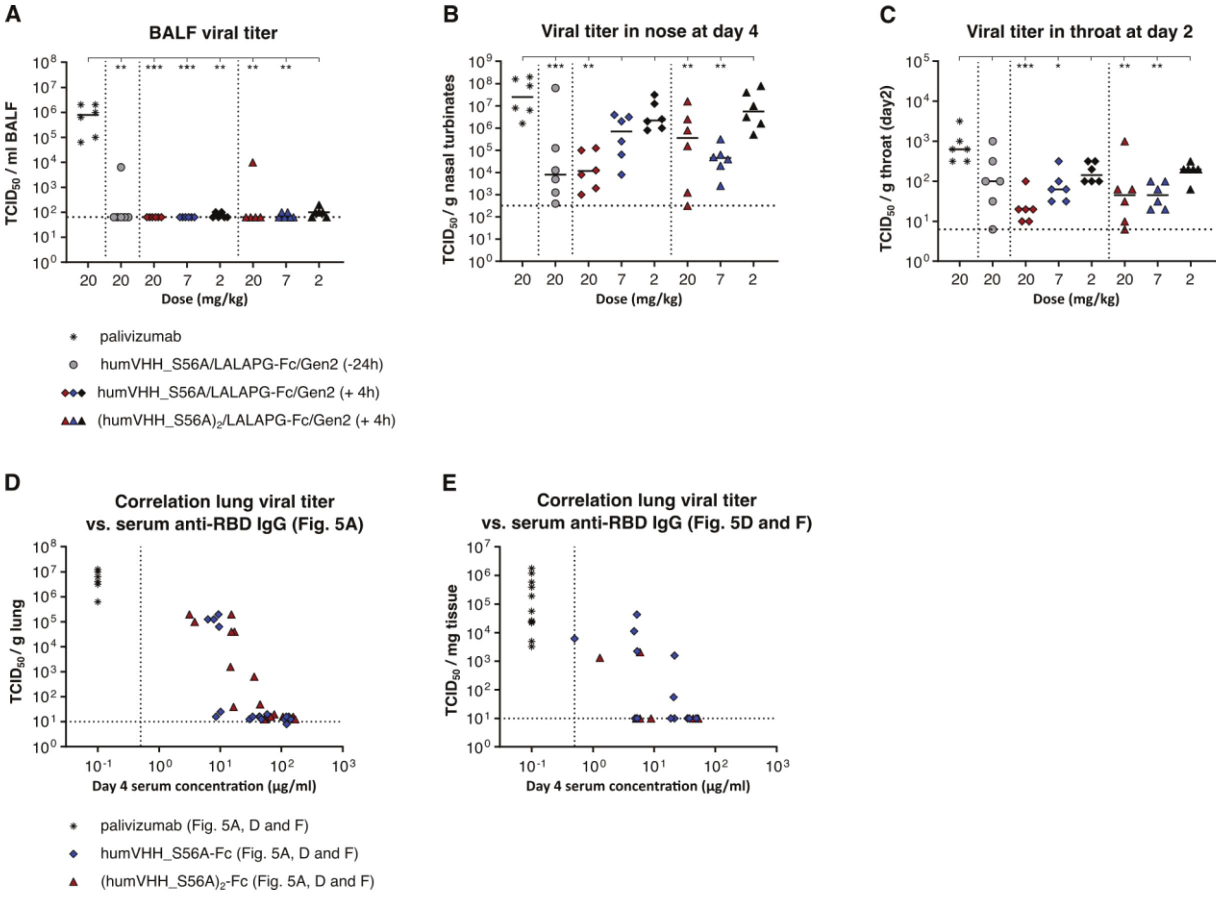

Fig. S13. Therapeutic administration of VHH72-Fc constructs reduces the SARS-CoV-2 viral burden in broncho-alveolar lavage fluid (BALF), nose and throat of Syrian hamsters. (**A to C**) Hamsters were challenged with $1x10^4$ PFU of BetaCoV/Munich/BavPat1/2020 and 4 hours later injected intraperitoneally with 20, 7, or 2 mg/kg of humVHH_S56A/LALAPG-Fc/Gen2 or $(humVHH\_S56A)_2$/LALAPG-Fc/Gen2. The negative control group was treated with 20 mg/kg of palivizumab, injected 4 hours after the challenge infection; hamsters in a prophylactic control group received 20 mg/kg of humVHH_S56A/LALAPG-Fc/Gen2 one day before the challenge. (**A and B**) Infectious virus titers in bronchoalveolar lavage (A) and nasal turbinates (B) were determined on day 4 after infection. (**C**) Viral titers were measured in the throat on day 2 after challenge. (**D and E**) Correlation between the serum concentration of RBD-binding antibodies and infectious virus in the lungs sampled on day 4 after challenge. Data in panels (**D**) and (**E**) are compiled from the therapeutic experiments shown in Fig. 5A or Fig. 5E and F, respectively. Hamsters were challenged with 1×10^4 PFU of BetaCoV/Munich/BavPat1/2020 and 4 hours later injected intraperitoneally with 20, 7, or 2 mg/kg of humVHH_S56A/LALAPG-Fc/Gen2 or $(humVHH\_S56A)_2$/LALAPG-Fc/Gen2 (D). Hamsters received an intraperitoneal injection of 7 mg/kg of humVHH_S56A/LALAPG-Fc/Gen2 one day prior to challenge or were treated by intraperitoneal injection of 1 or 7 mg/kg of humVHH_S56A/LALAPG-Fc/Gen2 or $(humVHH\_S56A)_2$/LALAPG-Fc/Gen2 16 hours after infection with 2×10^6 PFU of passage 6 BetaCov/Belgium/GHB-03021/2020 (E). Data were analyzed with the Mann-Whitney U-test. *, $p< 0.05$; **, $p<0.01$; ***, $p<0.001$; ****, $p<0.0001$.

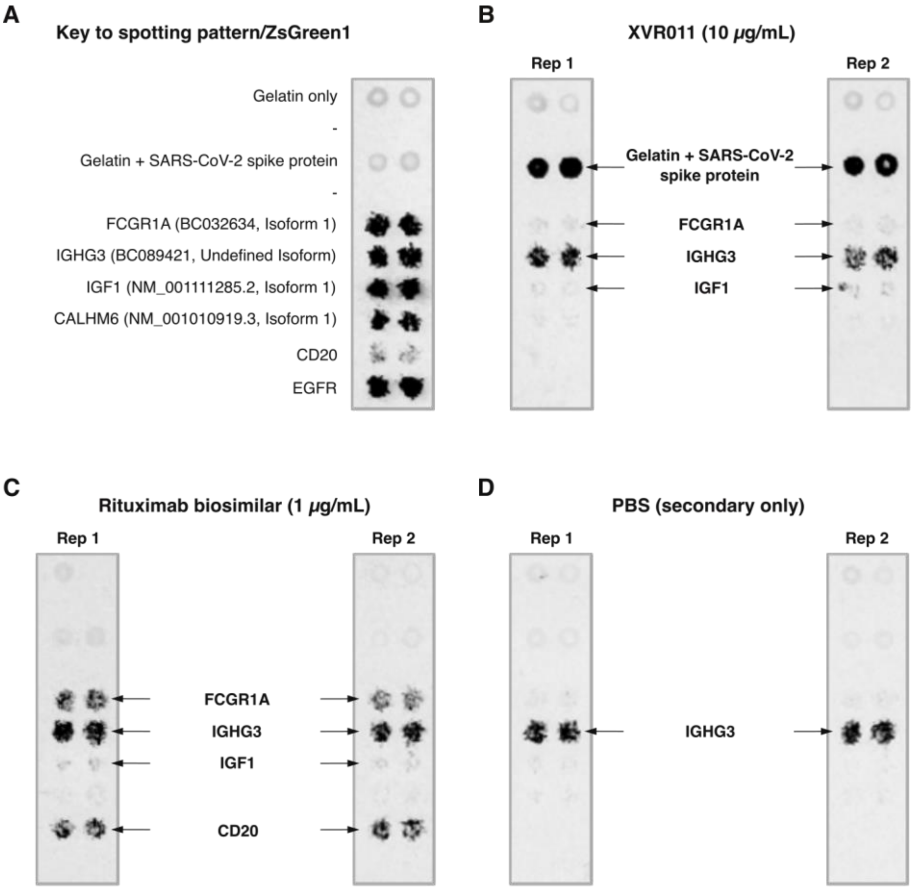

Fig. S14. HumVHH_S56A/LALA-Fc/Gen2 (= XVR011) is not poly-reactive. XVR011 at 10 µg/ml was assessed for binding against 5475 human plasma membrane proteins and cell surface tethered secreted proteins plus 371 human heterodimers expressed in transfected HEK293 cells. The confirmation screen for the initial hits of the library screen is shown. (**A**) Key to the microarray spotting pattern. Gelatin only and gelatin + SARS-CoV-2 spike protein were spotted. The other spots represent cells that were transfected with an expression plasmid for ZsGreen1 and the indicated respective cDNAs. (**B**) Reactivity of XVR011 at 10 µg/ml is shown by Alexa Fluor 647 anti-human IgG Fc antibody labeling, followed by fluorescence imaging. (**C**) Reactivity of Rituximab 1 µg/ml with the microarray with detection as in (**B**). (**D**) Reactivity with the microarray of the secondary antibody (PBS instead of primary antibody). Rep, replicate.

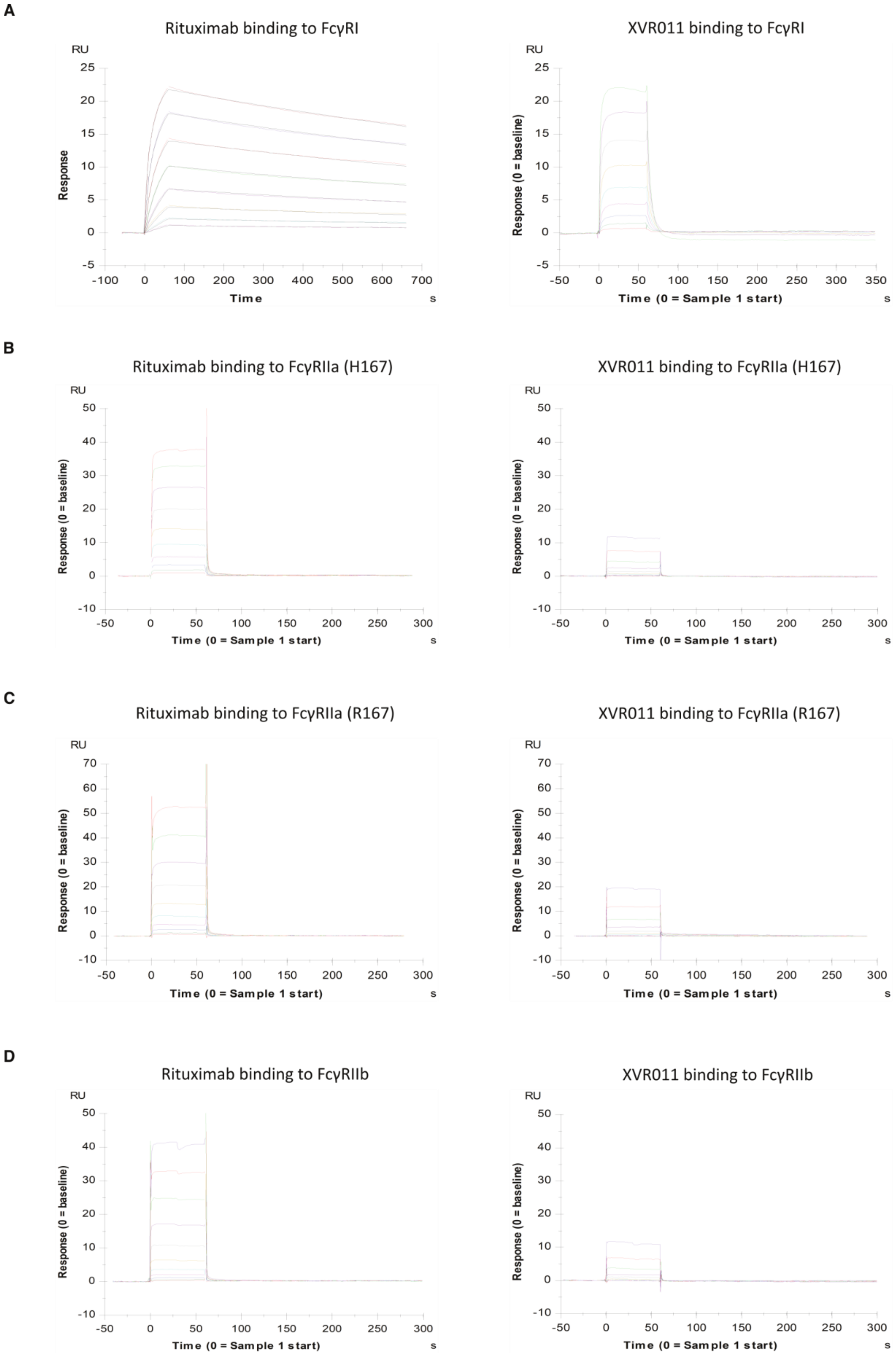

Fig. S15 (continued).

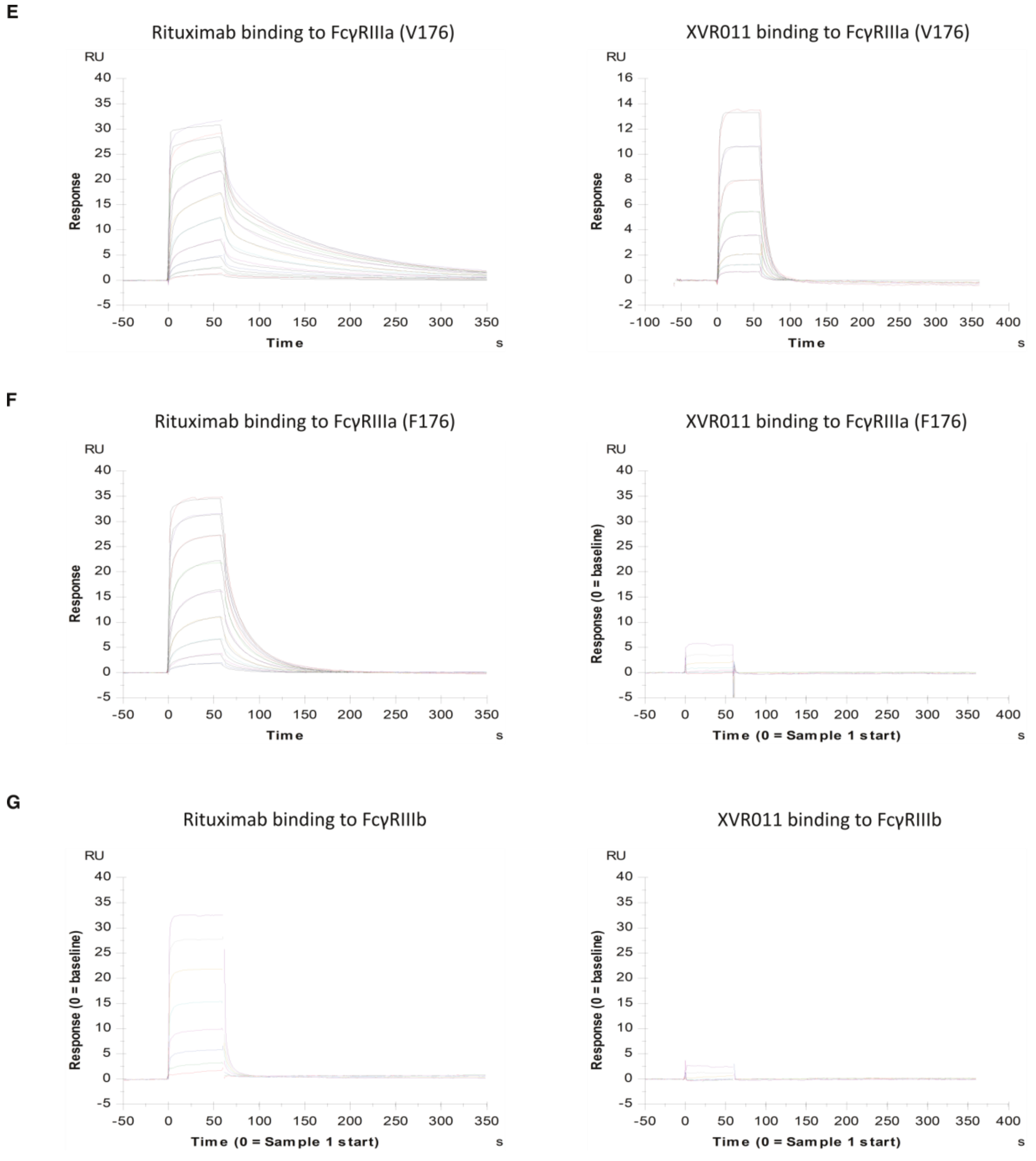

Fig. S15. Surface plasmon resonance (SPR) sensorgrams for the binding of XVR011 and rituximab to immobilized Fcγ receptors. Binding of rituximab (left) and XVR011 (right) to (**A**) FcγRI, (**B**) FcγRIIa (H167), (**C**) FcγRIIa (R167), (**D**) FcγRIIb, (**E**) FcγRIIIa (V176), (**F**) FcγRIIIa (F176), and (**G**) to FcγRIIIb are shown.

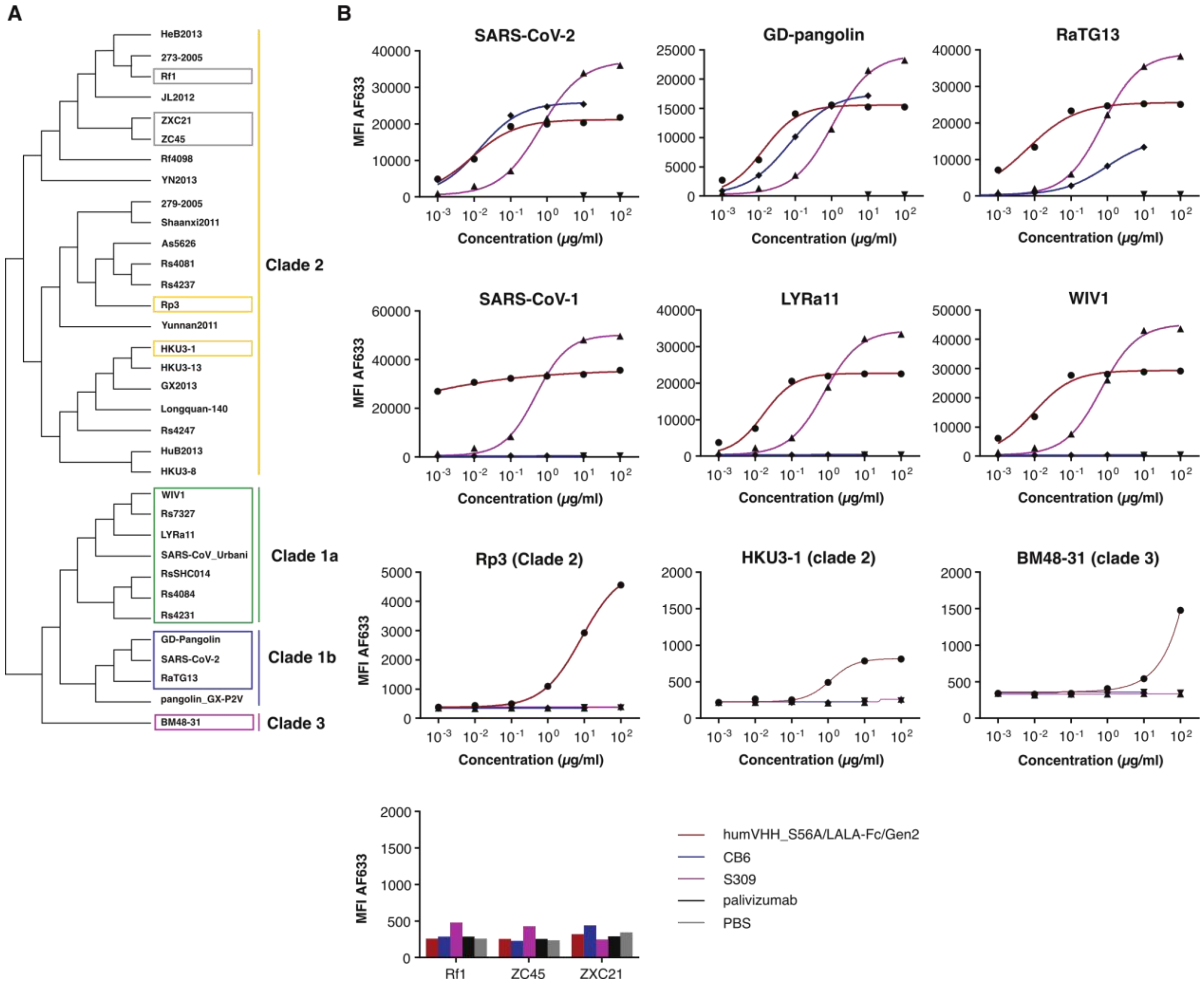

Fig. S16. HumVHH_S56A/LALA-Fc/Gen2 binds to the RBD of a diverse range of Sarbecoviruses. (**A**) Cladogram (unweighted pair group method with arithmetic mean [UPGMA] method) based on the RBD of SARS-CoV-1-related, SARS-CoV-2-related and clade 2 and clade 3 Bat SARS-related Sarbecoviruses. The colored boxes indicate the RBD variants that are bound by humVHH_S56A/LALA-Fc/Gen2 as determined by flow cytometry of either yeast cells that display the indicated RBD variants or HEK293T cells that express SARS-CoV-1 spike proteins in which the RBD is substituted by the indicated RBD variants. The gray boxes indicate the RBD variants for which no binding of humVHH_S56A/LALA-Fc/Gen2 could be observed. (**B**) Analysis of the binding of humVHH_S56A/LALA-Fc/Gen2, S309, CB6, and palivizumab to *Saccharomyces cerevisiae* cells that display the RBD of the indicated Sarbecoviruses. The graphs show the MFI of Alexa Fluor 633-conjugated anti-human IgG that was used to detect the binding of dilution series of the tested antibodies to *S. cerevisiae* cells that express the RBD derived from the indicated Sarbecoviruses.

Table S1. Overview of the VHH72-Fc constructs and their $PRNT_{50}$ SARS-CoV-2 neutralization values.

#	Short name	VHH72-Fc variant	$PRNT_{50}$ (µg/ml)
1	WT-VHH/12GS-WT-Fc	VHH72-GS(G_4S)$_2$-hIgG1hinge-hIgG1Fc	0.68
2	WT-VHH/WT-Fc	VHH72-GS-hIgG1hinge-hIgG1Fc	1.01
3	WT-VHH/LALAPG-Fc	VHH72-GS-hIgG1hinge-hIgG1Fc_LALAPG	0.57
4	humVHH/WT-Fc	VHH72_h1-GS-hIgG1hinge-hIgG1Fc	0.94
5	(humVHH)$_2$/WT-Fc	VHH72_h1-(G_4S)$_3$-VHH72_h1-GS-hIgG1hinge-hIgG1Fc	0.10
6	humVHH/LALAPG-Fc	VHH72_h1-GS-hIgG1hinge-hIgG1Fc_LALAPG	1.22
7	humVHH_S56A/WT-Fc	VHH72_h1_S56A-GS-hIgG1hinge-hIgG1Fc	0.12
8	humVHH_S56A/LALAPG-Fc	VHH72_h1_S56A-GS-hIgG1hinge-hIgG1Fc_LALAPG	0.13
9	humVHH/LALA-Fc/Gen2	VHH72_h1_E1D-(G_4S)$_2$-hIgG1hinge_EPKSCdel-hIgG1Fc_LALA_K447del	ND
10	humVHH_S56A /LALAPG-Fc/Gen2	VHH72_h1_E1D_S56A-(G_4S)$_2$-hIgG1hinge_EPKSCdel-hIgG1Fc_LALAPG_K477del	0.11
11	(humVHH_S56A)$_2$/LALAPG-Fc/Gen2	VHH72_h1_E1D_S56A-(G_4S)$_3$-VHH72_h1_E1D_S56A-GS-hIgG1hinge_EPKSCdel-hIgG1Fc_LALAPG_K477del	0.02
12	humVHH_S56A/LALA-Fc/Gen2	VHH72_h1_E1D_S56A-(G_4S)$_2$-hIgG1hinge_EPKSCdel-hIgG1Fc_LALA_K477del	0.09

PRNT: plaque reduction neutralization assay
ND: not determined.
Design 12 (humVHH_S56A/LALA-Fc/Gen2) was chosen for the development of the drug candidate that was cGMP manufactured and formulated as XVR011.

Table S2. **Apparent binding affinity of VHH72-Fc variants to immobilized mouse Fc-fused SARS-CoV-2 RBD (RBD-mFc).** Apparent kinetics of the 2:2 interaction is based on a global 1:1 fit of the replicate (n = 2) data; values are the averages of the replicates.

#	VHH-Fc variant	Apparent K_D (M)	Apparent k_{on} (1/Ms)	Apparent k_{off} (1/s)
5	$(humVHH)_2$/WT-Fc	<1.0E-12	2.65E+06	<1.0E-07
10	humVHH_S56A /LALAPG-Fc/Gen2	4.08E-11	1.52E+06	6.16E-05
11	$(humVHH\_S56A)_2$/LALAPG-Fc/Gen2	<1.0E-12	2.48E+06	<1.0E-07

Table S3. Peptide mapping results of the different VHH72-Fc constructs. LysC-Trypsin codigested protein was analyzed with LC-MS/MS, and peptides and peptide modifications detected with BioPharma Finder.

**#**	**VHH-Fc variant**	**Sequence coverage**	**Modifications**			
			Pyro-Glu	N-glycosylation	Glycation	Deamidation
2	WT-VHH/WT-Fc	84.4%	Q1 (98.13%)	N209	K238	N227
3	WT-VHH/LALAPG-Fc	82.5%	Q1 (92.28%)	N209	K160, K200	N227
4	humVHH/WT-Fc	83%	/	N209	K160, K238	N227
6	humVHH/LALAPG-Fc	83.3%	/	N209	K160, K238	N227
7	humVHH_S56A/WT-Fc	85.2%	/	N209	K238	N227
8	humVHH_S56A /LALAPG-Fc	84.7%	/	N209	K160, K238	N227
10	humVHH_S56A /LALAPG-Fc/Gen2	83.9%	/	N212	/	N230
12	humVHH_S56A /LALA-Fc/Gen2	80.1	/	N212	/	N230

Table S4. **Expression yields of VHH72-Fc constructs in transiently transfected ExpiCHO cells.**

#	VHH-Fc variant	ExpiCHO-S yield (mg/l)
1	WT-VHH/12GS-WT-Fc	638
2	WT-VHH/WT-Fc	403
3	WT-VHH/LALAPG-Fc	773
4	humVHH/WT-Fc	768
5	$(humVHH)_2$/WT-Fc	803
6	humVHH/LALAPG-Fc	1033
7	humVHH_S56A/WT-Fc	1215
8	humVHH_S56A /LALAPG-Fc	806

Table S5. Aggregation propensity of VHH72-Fc fusion proteins in PBS. SEC-MALS analysis of purified VHH72-Fc samples is shown. The peak corresponding to the molar weight of an assembled bivalent VHH-Fc protein was indicated as 'monomer peak'. Peak quantitation (%) is based on the refraction signal. HMW = high molecular weight species; LMW = low molecular weight species. *Molar mass as predicted by the ExPASy ProtParam tool.

**ID**	**VHH72-Fc variant**	**Quantitative analysis of total profile**					**Qualitative analysis of monomer peak**	
		Peak quantitation (%)					Molar mass (kDa)	
		Monomer	Multimer	HMW	LMW	Aggregates	Predicted*	MALS
2	WT-VHH/WT-Fc	97.8	0.5	0.2	1.2	0.2	79.7	79.3
3	WT-VHH/LALAPG-Fc	98.4	0.4	0.1	1.0	0.1	79.4	78.2
4	humVHH/WT-Fc	98.8	0.4	0.0	0.9	0.0	79.7	78.9
6	humVHH/LALAPG-Fc	98.9	0.6	0.0	0.5	0.0	79.5	77.9
7	humVHH_S56A/WT-Fc	97.4	0.6	0.2	1.7	0.1	79.7	78.8
8	humVHH_S56A /LALAPG-Fc	97.2	0.4	0.4	1.9	0.2	79.5	77.7

Table S6. Bivalent and tetravalent VHH72-Fc constructs occur as >96% monomers upon 10 days stress testing at 40°C in PBS without formulation. SEC-MALS analysis of purified VHH72-Fc samples is shown. The peak corresponding to the molar weight of an assembled bivalent VHH-Fc protein was indicated as 'monomer peak'. Peak quantitation (%) is based on the refraction signal. Qualitative analysis of the monomer peak was performed on the 200 µl peak elution fraction, as indicated in black in Fig. 3E. Values reported are the averages of duplicate measurements of two independently stress tested samples. n, number of samples; HMW, high molecular weight species; LMW, low molecular weight species; agg, aggregates. *Molar mass as predicted by the ExPASy ProtParam tool.

**Sample**				**Quantitative analysis of total profile**										**Qualitative analysis of monomer peak**		
**#**	**VHH72-Fc variant**	**Days at 40°C**	**n**	**Peak quantitation (%)**					**Polydispersity**					Molar mass (kDa)		Hydrodynamic radius rh(Q)w (nm)
				Monomer	Multimer	HMW	LMW	Agg	Monomer	Multimer	HMW	LMW	Agg	Predicted*	MALS	
**10**	humVHH_S56A /LALAPG-Fc/Gen2	0	2	98.1	0.2	0.6	0.9	0.3	1.006	1.297	1.288	1.536	1.964	79.1	77.9	4.3
		10	2	98.7	0.4	0.1	0.9	0.0	1.003	1.972	1.990	13.247	1.989		77.7	4.1
**11**	(humVHH_S56A)$_2$/LALAPG-Fc/Gen2	0	2	97.5	1.4	0.5	0.6	0.2	1.007	1.160	1.202	3.380	1.366	107.2	104.3	5.3
		10	2	95.1	2.6	0.6	1.7	0.1	1.008	1.187	1.485	15.592	1.454		104.7	5.1
**9**	humVHH/LALA-Fc/Gen2	0	2	98.2	0.5	0.3	1.2	0.0	1.011	2.218	2.579	2.465	1.554	79.2	79.3	4.3
		10	2	95.1	1.5	0.7	2.7	0.2	1.005	1.249	2.646	2.251	1.896		78.8	4.3
**12**	humVHH_S56A/LALA-Fc/Gen2	0	2	97.9	0.6	0.3	1.1	0.2	1.017	1.734	2.106	1.902	7.982	79.1	79.3	4.5
		10	2	96.1	1.1	0.6	2.1	0.1	1.003	1.172	2.069	2.345	1.922		78.8	4.3

Table S7. **Biophysical characteristics of bivalent versus tetravalent VHH72-Fc variants.** A greater hydrophobic interaction chromatography (HIC) retention time indicates increased apparent hydrophobicity. Solubility at increasing polyethylene glycol (PEG) concentrations was determined in two buffer systems (PBS pH 7.4 and 50 mM histidine, 250 mM proline, pH 5.5), and the midpoint of the sigmoidal PEG dose-response fit is indicated as '% midpoint'. A higher PEG midpoint score suggests greater solubility at high concentrations. Approximately 90% of the tetravalent sample formulated in 50 mM histidine, 250 mM proline pH 5.5 buffer aggregated at the lowest PEG3350 concentration (7.7 %), therefore a midpoint could not be determined. The isoelectric point (pI) was determined by capillary isoelectric focusing (cIEF). *Theoretical pI as calculated using the MPCT tool.

**Short name**	**VHH72-Fc variant**	**HIC**	**PEG assay (% midpoint)**		**pI**	
		retention time (min)	PBS pH 7.4	His/Pro pH 5.5	Theoretical*	cIEF
humVHH_S56A/WT-Fc	VHH72_h1_E1D_S56A-$(G_4S)_2$-hIgG1hinge-hIgG1Fc	19.9	15.2	11.3	6.0	6.3
humVHH_S56A/LALA-Fc	VHH72_h1_E1D_S56A-$(G_4S)_2$-hIgG1hinge-hIgG1Fc_LALA	19.8	14.8	10.7	6.0	6.3
humVHH_S56A/LALAPG-Fc	VHH72_h1_E1D_S56A-$(G_4S)_2$-hIgG1hinge-hIgG1Fc_LALAPG	19.8	15.1	11.4	6.0	6.3
$(humVHH\_S56A)_2$/WT-Fc	VHH72_h1_E1D_S56A-$(G_4S)_3$-VHH72_h1_S56A-GS-hIgG1hinge-hIgG1Fc	23.9	12.7	<7.7	5.5	5.9
$(humVHH\_S56A)_2$/LALA-Fc	VHH72_h1_E1D_S56A-$(G_4S)_3$-VHH72_h1_S56A-GS-hIgG1hinge-hIgG1Fc_LALA	23.7	12.1	<7.7	5.5	5.9
$(humVHH\_S56A)_2$/LALAPG-Fc	VHH72_h1_E1D_S56A-$(G_4S)_3$-VHH72_h1_S56A-GS-hIgG1hinge-hIgG1Fc_LALAPG	23.8	12.6	<7.7	5.5	5.9

Table S8. **Binding affinity of XVR011 and Rituximab to immobilized recombinant human Fcγ receptors determined by SPR.**

Fcγ receptor	XVR011 K_D (M) mean ± SD (n=3)	Rituximab K_D (M) mean ± SD (n=3)
FcγRI	1.76 ± 0.01 x 10^{-6}	K_{D1}: 7.73 x 10^{-10}; K_{D2}: 4.76 ± 0.27 x 10^{-9}*
FcγRIIa (H167)	> 20 μM	1.28 ± 0.14 x 10^{-6}
FcγRIIa (R167)	> 20 μM	2.90 ± 0.30 x 10^{-6}
FcγRIIb	> 20 μM	5.00 ± 0.43 x 10^{-6}
FcγRIIIa (V176)	5.66 ± 0.15 x 10^{-6}	1.43 ± 0.11 x 10^{-7}
FcγRIIIa (F176)	> 20 μM	6.98 ± 0.79 x 10^{-7}
FcγRIIIb	> 20 μM	1.76 ± 0.11 x 10^{-6}

* Estimated equilibrium dissociation constants (K_{D1} an K_{D2}) of rituximab interacting with FcγRI, fitted to a heterogenous ligand model.

All other data were fitted to a 1:1 binding model.
